# New criteria for efficient Raman and Brillouin amplification of laser beams in plasma

**DOI:** 10.1038/s41598-020-76801-z

**Published:** 2020-11-16

**Authors:** R. M. G. M. Trines, E. P. Alves, E. Webb, J. Vieira, F. Fiúza, R. A. Fonseca, L. O. Silva, R. A. Cairns, R. Bingham

**Affiliations:** 1 Central Laser Facility, STFC Rutherford Appleton Laboratory, Didcot, OX11 0QX UK; 2 GoLP/IPFN, Instituto Superior Técnico, Universidade de Lisboa, 1049-001 Lisbon, Portugal; 3 SLAC National Accelerator Laboratory, Menlo Park, CA 94025 USA; 4 ISCTE, Instituto Universitário de Lisboa, 1649-026 Lisbon, Portugal; 5 University of St Andrews, St Andrews, Fife KY16 9SS UK; 6 SUPA, Department of Physics, University of Strathclyde, Glasgow, G4 0NG UK

**Keywords:** Laser-produced plasmas, Nonlinear optics

## Abstract

Raman or Brillouin amplification of a laser beam in plasma has long been seen as a way to reach multi-PW powers in compact laser systems. However, no significant plasma-based Raman amplification of a laser pulse beyond 0.1 TW has been achieved in nearly 20 years, and only one report of Brillouin amplification beyond 1 TW. In this paper, we reveal novel non-linear criteria for the initial seed pulse that will finally open the door to efficient Raman and Brillouin amplification to petawatt powers and Joule-level energies. We show that the triple product of the coupling constant $\Gamma$, seed pulse duration $\tau$ and seed pulse amplitude *a* for the Raman seed pulse (or $a^{2/3}$ for Brillouin) must exceed a specific minimum threshold for efficient amplification. We also analyze the plasma-based Raman and Brillouin amplification experiments to date, and show that the seed pulses used in nearly all experiments are well below our new threshold, which explains the poor efficiency obtained in them. Finally, we analyze a recent Brillouin amplification experiment that used increased seed pulse power to obtain Joule-level amplification, and find excellent agreement with our theory.

## Introduction

Compression and amplification of laser pulses via Raman scattering is a well-known and successful technique in fibre optics^1^. Plasma-based compression and amplification of laser pulses via Raman or Brillouin scattering has been proposed to overcome the intensity limitations posed by solid-state optical systems^2–8^. Raman amplification in plasma has many advantages over amplification in solid media: (i) much higher peak intensities, so the same power can be reached using a much smaller and cheaper system (compare plasma-based particle acceleration^9^), (ii) the non-linear Raman process increases the bandwidth of the growing seed pulse, allowing for much shorter pulse duration than available via linear compression of the usually narrowband pump pulse, (iii) Raman amplification can access wave length ranges and pulse durations not easily available via solid-state systems, e.g. pulses of picosecond duration and petawatt power at 351 nm^10^.

In this paper, we perform the first detailed and systematic study of the full evolution of the seed and pump pulse, from the linear into the non-linear regime, for both Raman and Brillouin amplification, and derive new non-linear matching criteria for the optimal dimensions (amplitude and duration) of the initial seed pulse before, during and after the interaction [Eqs. (4) and (7) in the Theory section]. We focus on the dimensions of the seed pulse rather than its complete envelope because the full envelope is much harder to control (before the interaction) or diagnose (after the interaction) than the seed pulse amplitude and duration. We will show that our new criteria have “attractor” properties: if the seed pulse does not obey them initially, it will reshape itself until it does, and only amplify after that. These new criteria can be exploited to guide the design of future experiments and maximize their efficiency.

**Figure 1:**
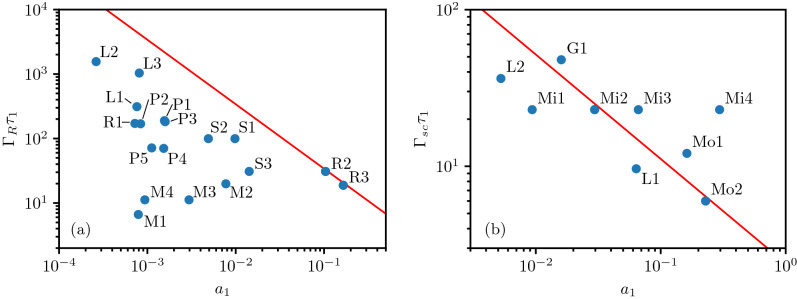
(**a**) Initial seed pulse parameters for past Raman amplification experiments performed at Princeton University (P), Livermore National Lab (L), Strathclyde University (S), or miscellaneous labs (M). Data are taken from Supplementary Table I in the Supplementary Information^27^. Shown is $\Gamma _R\tau _1$ versus $a_1$, where $\Gamma _R$ is the Raman backscattering coupling coefficient while $\tau _1$ and $a_1$ are the signal pulse’s duration and amplitude. All points represent input seed pulses to their respective experiments, except points R2 and R3. The points R1, R2 and R3 correspond to the pulse in the Ren experiment^13^ before, during and after amplification. The line $\Gamma _R \tau _1 a_1 = 3.4$ is shown in red. While no experiment has an initial seed pulse even close to the ideal line, the amplified pulses in the Ren experiment (R2, R3) are on the ideal line, indicating that this experiment may have achieved non-linear Raman amplification. (**b**) Initial seed pulse parameters for past Brillouin amplification experiments performed at LULI Laboratory (L), Rutherford Appleton Laboratory (G) and from the Marquès experiment (M)^18^. Data are taken from Supplementary Table II in the Supplementary Information^27^. Shown is $\Gamma _{sc}\tau _1$ versus $a_1$, where $\Gamma _{sc}$ is the strong-coupling Brillouin backscattering coupling coefficient. All points represent input seed pulses to their respective experiments, except points Mo1, Mo2. Mi1–Mi4: initial seed pulses from Ref.^18^, Mo1 and Mo2: output seed pulses from Ref.^18^ obtained for a 40 mJ input pulse (pulse Mi2). The line $\Gamma _{sc} \tau _1 a_1^{2/3} = 2.40$ is shown in red. We see that initial pulses Mi2 and Mi3, which provided the most efficient amplification, are closest to the ideal line, while output pulses Mo1 and Mo2 follow the ideal line closely.

Since 2000, there have been many experiments on Raman or Brillouin amplification in plasma by many groups^11–17^. Except for one recent Brillouin amplification experiment^18^, these did not lead to the true breakthrough in plasma-based Raman amplification that was promised by the early experiments at Princeton University^12,13,19,20^. In this paper, we will argue that there are two main reasons for this. First, Raman amplification is most efficient in the non-linear regime, characterised by full pump depletion, and seed pulse compression as well as amplification^6,21,22^ (see below). However, almost all experiments to date use low-power seed pulses that are in the inefficient linear regime (no pump depletion or seed pulse compression), and will not (fully) reach the non-linear regime within the limited interaction distance available in plasma-based amplification. (As elucidated in the Supplementary Information, the interaction distance needed to reach the non-linear regime in the experiments of Refs.^11–13,15,17^ is longer than the length of the plasma column provided in those.) Second, the main quantity of merit for a Raman laser amplifier should be the absolute power or energy of the amplified seed pulse^6^. However, in nearly every experiment on Raman amplification the focus is on the “gain factor” instead, which is the energy of the amplified seed pulse divided by the energy of the initial seed pulse^7,15–17,23^. “Gain” is a useful concept in crossed-beam energy transfer between two laser beams of comparable size^24^. In Raman or Brillouin amplification, where the seed pulse is often much weaker than the pump, its use is fraught with problems. Not only does the use of “gain” encourage a reduction in input seed pulse power and a matching reduction in absolute output power^7,16^ to artificially boost the “gain”, but it also leads the experiments away from efficient plasma-based Raman or Brillouin amplification, for which seed pulses with more energy, not less, are needed^25^. And indeed, recent experiments have seen a marked decrease in initial seed pulse energy, power and intensity^16,17,23^ compared to their predecessors by the same groups^14,15,26^, i.e. the opposite of what is needed for more efficient amplification, as demonstrated in this paper.

Below, we show that the triple product $\Gamma _R \tau _1 a_1$ for an optimized nonlinear seed pulse satisfies $\Gamma _R \tau _1 a_1 \approx 3.4$, where $\Gamma _R$ is the Raman scattering coupling constant while $\tau _1$ and $a_1$ denote the duration and amplitude of the initial seed pulse [see Eq. (4) below]. Once the triple product has attained its optimal value, it will stay there throughout the amplification process. However, the further the triple product for the initial seed pulse is from the ideal value, the longer it will take to get there. This causes problems in plasma-based amplification experiments where the interaction distance is often limited: (i) the ideal value may never be reached within the available interaction length, and (ii) even if the ideal value is reached, the efficiency of the amplification process is often poor when the initial value of the triple product is too far from the ideal. We will show that efficient amplification over a limited distance can be achieved by using more powerful seed pulses that are non-linear from the start, i.e. $\Gamma _R \tau _1 a_1 \sim 3.4$, while low efficiency will be obtained if the initial seed pulse is only linear, i.e. $\Gamma _R \tau _1 a_1 \ll 3.4$, and “negative” amplification (energy flowing from seed to pump) is found for $\Gamma _R \tau _1 a_1 \gg 3.4$. To motivate our research, we first show, in Fig. 1a, a survey of $\Gamma _R\tau _1$ versus $a_1$ for the initial seed pulse for every significant Raman amplification experiment since 2000, as well as the ideal curve $\Gamma _R \tau _1 a_1 = 3.4$ (in red). The data displayed in this figure are given in Supplementary Tables I and II in the Supplementary Information^27^. We note that, for nearly every experiment, the initial seed pulses display $\Gamma _R \tau _1 a_1 \ll 3.4$, well short of our new criterion for efficient nonlinear amplification.

Contrary to the input pulses, the output pulse duration was only provided by three experiments: P3, P4^12,20^ (output pulses of these not shown in Fig. 1) and the Ren experiment^13^ (output pulses R2, R3 are shown in Fig. 1). From this, we could estimate output values for $\Gamma _R \tau _1 a_1$: $\sim 3.1$, $\sim 2.8$ and $\sim 3.7$ for experiments P3, P4 and Ren (R2–R3), respectively^27^. This suggests that these experiments did reach the non-linear regime. In most other experiments, the output duration is not provided, although the narrowing of the seed pulse spectrum seen in a number of those^16,17^ suggests seed pulse stretching instead of shortening, indicative of linear amplification.

Further analysis of experimental output pulses also revealed that (i) in experiments with weak seed pulses, non-linear amplification only happens in the centre of the seed pulse and not in the wings, resulting in a strong reduction in transverse spot diameter which severely limits the overall energy efficiency^12,13^, while the strong seed pulses used for Brillouin amplification by Marquès et al.^18^ are not affected by spot size reduction (see also Supplementary Table III in the Supplementary Information^27^), and (ii) in most Raman experiments, Raman backscattering from thermal noise contributes 10–30% of the measured output energy, which degrades output pulse quality. In support of these findings, we conducted a series of 2-dimensional particle-in-cell simulations of Raman amplification in which the initial seed pulse intensity was progressively reduced, leaving all other parameters invariant. Our simulations show that (i) reducing the input seed intensity by a factor 10 reduced the output seed intensity (after the same interaction length) by a factor $\sim \sqrt{10}$, (ii) since RBS from thermal noise is unaffected by the seed pulse intensity, it becomes relatively more important for low input seed intensities, leading to a reduction in output seed quality, (iii) for seed pulse intensities between $10^{12}$ and $10^{14}$ W/cm$^2$ (as used in most experiments) one observes a significant narrowing of the seed pulse spot diameter, as reported in two experiments^12,13^, and finally (iv) the amplification of seed pulses with high initial intensities appears to happen quickly enough to outrun the growth of deleterious transverse instabilities like filamentation, so such seed pulses are relatively less affected by e.g., filamentation than low-intensity seed pulses. This emphasizes the need to use a strong seed pulse satisfying our new criteria in a Raman or Brillouin amplification experiment, to ensure that true seed amplification dominates over noise amplification and that nonlinear amplification is achieved across a wide spot, not just near the axis of propagation. We also stress that, while Raman backscattering is “in principle” a 1-D instability, there are various competing 2-D instabilities and other 2-D effects that one must also consider when amplifying a realistic seed pulse. A rigid separation between one-dimensional and multi-dimensional theory will not work here. Details of both the analysis of experimental output pulses and the series of 2-D simulations are given in the Supplementary Information^27^.

We thus find that the new criterion $\Gamma _R \tau _1 a_1 = 3.4$ has three distinct purposes: (i) it can be used to design the proper seed pulse before the interaction, (ii) it can be used to predict which pulse shapes can and cannot be obtained via Raman amplification (since the final $a_1$ and $\tau _1$ are no longer independent), and (iii) it can be used after the interaction to prove that the seed pulse did indeed reach the non-linear regime. The latter is vital to distinguish true Raman amplification (both energy gain and seed pulse shortening^6,12^) from processes like “crossed beam energy transfer” (CBET, energy transfer without pulse shortening)^24,28^, continued backscattering by the pump beam long after the passage of the seed pulse^15,29^ or merely Raman backscattering from noise^30^. To date, no previous work on Raman or Brillouin amplification has provided such an easy, direct test for non-linearity of the amplification. Naturally, we can only apply our test to experiments that provide data on seed pulse duration in addition to pulse energy, so we need such data in every experiment to judge its true merit.

For strongly coupled Brillouin amplification, the ideal seed pulse satisfies a slightly different equation, $\Gamma _{sc} \tau _1 a_1^{2/3} \approx 2.40$ [see Eq. (7) below]. In Fig. 1b, we analyse four strongly coupled Brillouin amplification experiments since 2010^15,18,23,31^ in the same way as the Raman amplification experiments. Two early Brillouin experiments (Refs.^15,23^, input pulses represented by points L1 and L2) used weak seed pulses well below the ideal line for sc-Brillouin amplification (in red), and reported weak amplification. The Guillaume experiment (Ref.^31^, input pulse is point G1) reported promising amplification, but the interaction length was too short to unlock this experiment’s full potential. The recent Marquès experiment^18^ explored a range of initial seed pulse intensities, input pulses given by points Mi1 to Mi4, and showed good amplification for input seed pulses Mi2 and Mi3 (close to the ideal line), weak amplification for seed pulse Mi1 (too far below the line), and “negative” amplification for seed pulse Mi4 (too far above, causing energy to flow from the seed back into the pump). This experiment thus provides solid support for our model. It also demonstrates that the initial seed pulse should be strong, but not too strong, to avoid “negative” amplification. Points Mo1 and Mo2 represent two output pulses from this experiment, showing that the amplified pulses also tend to stay close to the ideal line identified here.

## Theory

In the context of Raman or Brillouin amplification, analytical models have been derived under the assumption that, at advanced interaction times $\Gamma t \gg 1$, the pump beam is fully depleted while the basic envelope shape of the asymptotic seed pulse does not change during amplification, and its amplitude and duration evolve according to well-defined scaling laws^6,7^. However, in an experiment with a fixed limited interaction length, this efficient non-linear “pump depletion” regime may or may not be reached, depending on the time it takes for the initial seed pulse to evolve into the correct asymptotic shape. To maximize the efficiency, the amplification process should enter this non-linear “pump depletion” regime as soon as possible, skipping the inefficient linear regime entirely and making the most of the limited interaction distance. The core idea of this paper is to demonstrate the need to force the amplification process into the non-linear regime by using an initial seed pulse that is shaped as if it is already non-linear. This leads to novel nonlinear matching conditions for this pulse (with “attractor” properties), in addition to the well-known linear matching conditions $\omega _0 = \omega _1 + \omega _2$ and $\text {k}_0 = \text {k}_1 + \text {k}_2$. Here we show how these novel criteria govern the evolution of the optimal seed pulse in Raman and Brillouin amplification. We demonstrate how the initial laser pulses need to be shaped to speed up the amplification process and improve its efficiency.

### Raman amplification

Raman amplification in plasma is governed by the following three-wave system^1,6,21,22^:

1 $$\begin{aligned} (\partial /\partial t \pm v_g \partial /\partial x) a_{0,1}&= \mp i\Gamma _R a_{1,0} b^{(*)}, \end{aligned}$$

2 $$\begin{aligned} (\partial /\partial t + 3v_e^2 (k/\omega _{pe}) \partial /\partial x) b&= -i\Gamma _R a_0 a_1^*. \end{aligned}$$

Here, $a_0$ and $a_1$ denote the scaled envelopes of pump and seed pulse respectively, $a_{0,1} \equiv 8.55\times 10^{-10} g^{1/2} (I_{0,1} \lambda _{0,1}^2 [\text {Wcm}^{-2} \upmu \text {m}^2])^{1/2}$, where $g=1$ ($g=1/2$) denotes linear (circular) polarisation. Let $\omega _0$, $k_0$ and $n_{cr}$ denote the pump laser frequency, wave number and critical density, $v_e = (k_B T_e/m_e)^{1/2}$ the electron thermal velocity, and $n_e$ and $\omega _{pe}$ the background electron density and corresponding plasma frequency. We define $b = \alpha _R \delta n_e/n_e$ where $\delta n_e$ is the plasma wave density fluctuation, $k_L \approx 2k_0 \approx 2\omega _0/c$ is the wave number of the plasma wave, $\alpha _R = g^{1/2} (\omega _{pe}/\omega _0)^{3/2}/2$ and $\Gamma _R = [\omega _0 \omega _{pe}/(4g)]^{1/2} = \omega _0 (n_e/n_{cr})^{1/4}/\sqrt{4g}$. The group velocity of the pump pulse is then $v_g = c^2 k_0/\omega _0 = c(1-n_e/n_{cr})^{1/2}$. This model remains valid as long as the pump amplitude remains below the wave breaking threshold: $||a_0|| < a_{wb} \equiv \alpha _R/\sqrt{2}$.

As explained in the Supplementary Information^27^, once the seed pulse amplitude $a_1$ exceeds the pump pulse amplitude $a_{00}$, the growing seed pulse solution to (1)–(2) will take on a “$\pi$-pulse” shape, where height and duration of the first peak after interaction time *t* are given by $||a_1||(t) = (2A/\xi _M) a_{00}^2 \Gamma _R t$, $\Gamma _R \tau _1(t) = \xi _M \Delta \xi /(2 a_{00}^2 \Gamma _R t)$, with $A \approx 1.29$, $\Delta \xi \approx 2.65$, while $5< \xi _M < 7$ in relevant cases^6,32^. Using a pump pulse duration of $\tau _0 = 2t$ (for interaction time *t*, the counter-propagating seed pulse sees 2*t* of pump pulse), we immediately find two novel non-linear matching conditions for efficient pulse amplification:

3 $$\begin{aligned} \Gamma _R^2 a_{00}^2 \tau _0 \tau _1&= \xi _M\Delta \xi \approx 15, \end{aligned}$$

4 $$\begin{aligned} \Gamma _R ||a_1|| \tau _1&= A\Delta \xi \approx 3.4. \end{aligned}$$

The asymptotic energy transfer efficiency for the first peak is given by $\eta = ||a_1||^2 \tau _1(t)/(2 a_{00}^2 t) = A^2 \Delta \xi /\xi _M \approx 4.4/\xi _M$. Thus, $\eta$ is constant for a given configuration, and decreases with increasing $\xi _M$. We note that these relations require $a_1(0) > a_{00}$^27^, and are thus not applicable to very long seed pulses with very low amplitude.

These relations have not been recognized before, and have profound significance in optimizing the amplification process as well as designing the experimental setup to achieve desired pulse characteristics. Equation (3) allows one to derive scalings for the seed pulse duration $\tau _1(t)$ and amplitude $a_1(t)$, and also to tune these parameters via the intensity of the pump pulse^10^. Equation (4) provides a relationship between the duration and amplitude of a fully developed non-linear seed pulse, which does not depend on the pump pulse at all. This relation can be used to show that the amplified seed pulse did indeed reach (or at least approach) the efficient non-linear regime. It can also be used to obtain a deeper understanding of earlier work, as discussed in the Supplemental Material^27^. Even more importantly, Eq. (4) can be used for the tailoring of the initial seed pulse in experiments: $\tau _1(0)$ and $a_1(0)$ are not independent parameters, but should obey Eq. (4) to push the amplification into the pump depletion regime from the start and thus maximize efficiency. A non-optimal initial seed pulse will first reshape itself to become optimal before it can be amplified^10,33^, reducing the amplification efficiency after a given interaction length. This is particularly important for Raman or Brillouin amplification in plasma, where the interaction length is much more limited than in fibre optics^1^.

We confirm the non-linear matching conditions (3) and (4) in our simulations below. A full derivation of these conditions, as well as a discussions of their properties and of the influence of thermal effects ($v_e > 0$) is given in the Supplementary Information^27^.

The true strength of the relation (4) shows in many ways. (i) It remains valid for a wide range of pump and seed intensities, plasma densities, etc., as discussed below. (ii) It explains the transverse “horseshoe” shape of the amplified pulse, even when amplifying higher-order transverse pulse modes where the topology of pump and seed pulses is different^10,27,34,35^. (iii) It continues to hold well beyond the reach of the original three-wave envelope model, e.g. for a non-constant pump amplitude, or beyond the traditional wave-breaking limit, or in warm plasma, or for particle-in-cell computer simulations that do not know anything about three-wave models. This is needed to verify that the $\pi$-pulse shape for the seed pulse is still likely to be an attractor in practical situations, which is often stated but never proved^6,36^. We will explore this below and in the Supplementary Information^27^.

### Brillouin amplification

Brillouin amplification is similar to Raman amplification, only the Langmuir wave is replaced by a low-frequency ion-acoustic wave, so the laser beams can have (nearly) the same frequency. Brillouin amplification in the so-called weak-coupling regime^4,5,37^ can be treated in the same way as Raman amplification^27^. Here, we will focus on Brillouin amplification in the *strong-coupling* regime (sc-Brillouin), where the ion-acoustic plasma wave is a driven (by the beating between pump and seed pulses) rather than a resonant mode. In this regime, where $a_{00}^2 > 8g (\omega _0/\omega _{pe})^2 \sqrt{Zm_e/m_i} v_e^3/c^3$ (ions with mass $m_i$ and charge *Ze*), the equations for $a_{0,1}$ remain the same, while the equation for $b = \alpha _{sc} \delta n_e/n_e$ becomes^7,37^:

5 $$\begin{aligned} \partial ^2 b /\partial t^2 = -\Gamma _{sc}^2 a_0 a_1, \end{aligned}$$

with $\Gamma _{sc}^3 = (v_g/c)^2 \omega _{pi}^2 \omega _0/(2g) = \omega _0^3 (Zm_e/m_i) (n_0/n_{cr}) (1-n_0/n_{cr})/(2g) = 2\omega _s \Gamma _B^2$, $\alpha _{sc} = \omega _{pe}^2/(4\omega _0 \Gamma _{sc})$. For $a_1(t) > a_{00}$, and in similar fashion to our treatment of Raman amplification, we consider a quasi-$\pi$-pulse “attractor” solution for (1) and (5) that scales as $||a_1||(t) = A (a_{00}^2 \Gamma _{sc} t)^{3/4}$ and $\Gamma _{sc} \tau _1(t) = \Delta \xi /(a_{00}^2 \Gamma _{sc} t)^{1/2}$, with $\Delta \xi \approx 3.3$ and $A \approx 0.62$^7,27,38^. Again using $\tau _0 = 2t$, we find the following non-linear matching conditions^27^:

6 $$\begin{aligned} \Gamma _{sc}^3 a_{00}^2 \tau _0 \tau _1^2&= 2(\Delta \xi )^2 \approx 22, \end{aligned}$$

7 $$\begin{aligned} \Gamma _{sc} ||a_1||^{2/3} \tau _1&= A^{2/3} \Delta \xi \approx (13.8)^{1/3} \approx 2.40. \end{aligned}$$

The asymptotic efficiency for this case is $\eta = a_1^2(t)\tau _1(t)/(2a_{00}^2 t) = A^2 \Delta \xi /2 \approx 0.63$. The role of (6) and (7) matches that of (3) and (4) for Raman amplification. Like (3) and (4), (6) and (7) require $a_1(0) > a_{00}$^27^.

While the notion that $a_1^2 \Gamma _{sc}^3 \tau _1^3 = \text {const.}$ for a Brillouin-amplified seed pulse (or $a_1 \Gamma _R \tau _1 = \text {const.}$ for Raman) was advanced in our earlier work^39,40^ and later also by by Chiaramello et al.^41^, these papers do not provide accurate numerical values for those constants, while we do so here in Eqs. (4) and (7). This results in: (i) an accurate numerical criterion for both the design and the evolution of seed pulses for both Raman and Brillouin amplification, which can be tested against simulations, (ii) a practical numerical test to determine whether an amplified seed pulse in an experiment has reached the non-linear stage or not, and (iii) an analytical expression for the efficiency $\eta$, which can also be tested against simulations. The earlier work^39–41^ does not provide any of these, since it lacks accurate numerical coefficients.

### Effects of damping and chirp

Various phenomena can influence both the amplitude threshold for a viable seed pulse and the efficiency of the amplification process. Damping of the plasma wave and chirping of the frequency of either the pump beam or the plasma wave are the two most prominent. Both have been put forward as a means to suppress pump Raman backscattering from low-intensity noise while still allowing the amplification of a higher-intensity seed pulse^42,55^.

The effect of Landau damping on the triple product is discussed in detail in the Supplemental Information^27^. The effects of collisional damping^42,43^ or ionisation damping^44^ on the three-wave model for Raman amplification are broadly similar. It is well-known that damping imposes a threshold on the pump amplitude: ${\Gamma_{R}} a_{00} > \nu$ for Raman scattering or $\Gamma_{sc} a_{00}^{2/3} > \nu$ for sc-Brillouin scattering, with $\nu$ the damping coefficient^37^. Damping also introduces a threshold for the initial seed amplitude: $\Gamma_{R} a_1(0) > \nu$ for Raman, or $\Gamma _{sc} a_1(0)^{2/3} > \nu$ for sc-Brillouin^27^. A strong seed pulse with $a_1(0) > a_{00}$ will satisfy these requirements by default, while weak seeds from noise with $\Gamma_{R} a_1(0) < \nu$ will be damped away. This is how “quasi-transient” Raman amplification works^42^, see also Sections II.A and II.C of the Supplemental Information^27^. The efficiency $\eta$ of the process is also affected: $\tilde{\eta }= \eta \tilde{a}_{00}/a_{00} < \eta$ for Raman amplification^27^, as could be expected.

The effects of chirp (frequency detuning of the three-wave frequency matching condition $\omega _0 - \omega _1 - \omega _L = 0$) have been studied by Malkin et al.^55^ and Farmer et al.^45^. Such detuning can be caused by (i) a chirp in the pump frequency $\omega _0$^55^, (ii) a plasma density gradient, leading to a chirp in the Langmuir wave frequency $\omega _L$ via the plasma frequency $\omega _p$^55^, and (iii) a plasma temperature gradient, leading to a chirp in the Langmuir wave frequency $\omega _L$ via the thermal term in the Bohm-Gross dispersion relation $\omega _L^2 = \omega _p^2 + 3 v_T^2 k^2$^45^.

The effects of chirp on our work are as follows. Inspired by Ref.^55^ for Raman amplification, we define the chirp rate $q = c(\omega '_p - 2\omega '_0)$, where $\omega ' \equiv \partial \omega /\partial z$. For the Raman growth (Raman bandwidth) to outstrip the detuning, we need $\Gamma_R^2 a_{00}^2 > q$^46,55^. Chirp also introduces a threshold for the seed pulse: $\Gamma_R^2 a_1(0)^2 > q$^27^. In similar fashion to damping, chirp can thus be used to suppress the amplification of weak seeds from noise, while allowing the amplification of a strong seed with $a_1(0) > a_{00}$^6,55^. This applies to all types of chirp (pump frequency, density gradient, temperature gradient). From Ref.^55^, we find that the chirp rate *q* influences neither the triple product nor the value of $\xi _M$. Regarding the efficiency in the presence of chirp: the pump depletion in the presence of chirp equals $1 - [q/(\Gamma_R^2 a_{00}^2)]^2 \xi _M^4/[16(1+\xi _M)^2]$^55^. This implies full pump depletion for $q=0$, and reduced pump depletion for $q \not = 0$, although the efficiency reduction is modest for $q \ll \Gamma_R^2 a_{00}^2$. For sc-Brillouin amplification with chirp, we find in similar fashion that $\Gamma _{sc}^2 a_{00}^{4/3} > q$ and $\Gamma _{sc}^2 a_1(0)^{4/3} > q$. Further details can be found in e.g. the work by Lehmann and Spatschek^47,48^.

We note that Eq. (4) is vital in the derivation of the thresholds for the seed pulse amplitude^27^, which underlines the importance of this novel finding.

## Numerical simulations

### Scaling of seed pulse duration and amplitude

To verify the validity of Eqs. (4) and (7), we have carried out several one-dimensional particle-in-cell (PIC) simulations using the codes XOOPIC^49^ and OSIRIS^50^. The parameters of these simulations, covering a wide range of scenarios, are discussed at length in the “Methods” section.

**Figure 2:**
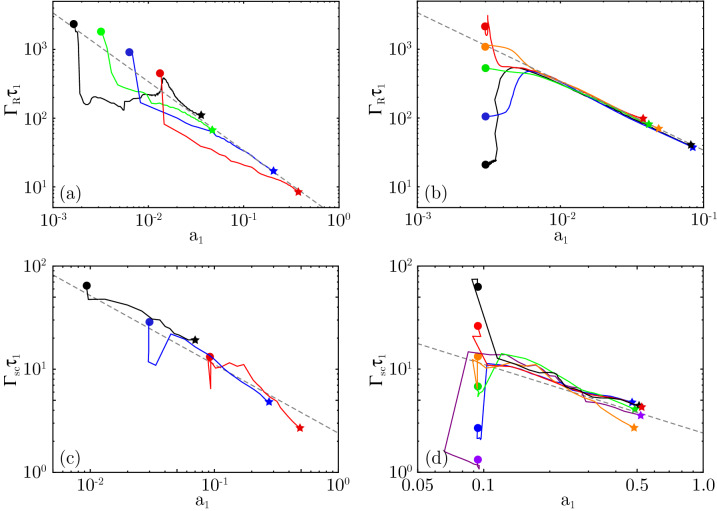
Evolution of scaled duration ($\Gamma _R\tau _1$ or $\Gamma _{sc}\tau _1$) versus peak amplitude ($a_1$) of cold-plasma Raman (**a**,**b**) and sc-Brillouin (**c**,**d**) amplified pulses for different pump amplitudes (**a**,**c**) or initial seed durations (**b**,**d**), demonstrating the attractor nature of the ideal solutions. (**a**) $n_0/n_{cr} = 0.0044$, and $a_0/a_{wb}=0.25$ (black), 0.5 (green), 1.0 (blue) and 2.0 (red) where $a_{wb} = 0.006086$, with initial seed amplitude $a_1 = a_0$ for each case. (**c**) $n_0/n_{cr} = 0.3$, and $a_0=0.0085$ (black), 0.027 (blue) and 0.085 (red); again, $a_1 = a_0$. (**b**) $n_0/n_{cr} = 0.0044$, $a_1 = a_0 =0.75 a_{wb}$. The initial durations of the seed pulse are $\tau _1/\tau _R = 0.02$ (black), 0.1 (blue) 0.5 (green), 1.0 (orange) and 2.0 (red). (**d**) $n_0/n_{cr} = 0.3$, and pump and seed amplitudes of $a_0 = a_1 = 0.085$. The initial durations of the seed pulse are $\tau _{1}/\tau _{B}=0.1$ (purple), 0.2 (blue), 0.5 (green), 1.0 (orange), 2.0 (red), and 5.0 (black). The dashed lines correspond to Eqs. (4) for Raman, and (7) for sc-Brillouin, respectively.

In Fig. 2, we show the evolution of $\Gamma _{R,sc}\tau _1$ versus $a_1$ for simulations of Raman in cold plasma (a,b) and sc-Brillouin (c,d) amplification. For each curve, the seed pulse evolution starts at the circle and finishes at the star. We observe that $\Gamma _R \tau _1 a_1$ and $\Gamma _{sc}\tau _1 a_1^{2/3}$ are not constants of motion for realistic seed pulses, but evolve towards asymptotic limits. The dashed lines in each frame represent Eqs. (4) and (7). Frames (a) and (c) show $\Gamma _{R,sc}\tau _1$ versus $a_1$ for various initial pump and seed pulse intensities, where $\tau _1(0) = \tau _R$ or $\tau _1(0) = \tau _B$ in each simulation. We find that the evolving seed pulses closely follow the predictions (4) and (7), irrespective of the pump intensity chosen in the simulations. Frames (b) and (d) show $\Gamma _{R,sc}\tau _1$ versus $a_1$ for fixed pulse intensities, while the initial pulse duration was moved away from the value required by the non-linear matching conditions (4) or (7). We find that in each case the seed pulse first evolves, with the duration adjusting and the amplitude staying nearly constant, until it obeys (4) or (7), respectively (this phase is called the “linear stage” of the amplification^6^). The pulse then amplifies as dictated by these criteria (with $a_1 \Gamma _R \tau _1$ sometimes performing several oscillations around the ideal value first). This specific behaviour was found in all our simulations, for all laser and plasma parameters we used. This demonstrates the following: (i) the $\pi$-pulse solution for Raman and its Brillouin equivalent are likely to be attractors, as predicted^6,7^, (ii) Eqs. (4) and (7) remain valid even in PIC simulations which go well beyond the approximations underlying the envelope models from which those equations are derived, and (iii) changing the initial seed pulse duration has no significant effect on the triple product $\Gamma _R\tau _1 a_1$ or $\Gamma _{sc} \tau _1 a_1^{2/3}$ of the end result, so $\tau _1(0)$ should not be treated as a free parameter, but set using (4) or (7) instead, to maximize efficiency.

Regarding the “wiggling” of the trajectories in Fig. 2: it can be shown that the evolution of $\Gamma _R \tau _1 a_1$ around its equilibrium value of 3.4 (or $\Gamma _{sc} \tau _1 a_1^{2/3}$ around 2.40) is not dissimilar to that of a weakly damped oscillator. Since the initial seed pulse shape is not equal to the asymptotic shape (this would not be manageable in an experiment either) we control the initial value of $\Gamma _R \tau _1 a_1$ but not its time derivative. It can thus be expected that $\Gamma _R \tau _1 a_1$ will execute a few oscillations around its equilibrium before it settles. This will also explain the behaviour of $\Gamma _{sc} \tau _1 a_1^{2/3}$ for the Marquès experiment, which started below the ideal line and finished above: there was not enough time for $\Gamma _{sc} \tau _1 a_1^{2/3}$ to settle. It also explains why initial seed pulses with $\Gamma _R \tau _1 a_1 = 3.4$ may amplify with less speed or efficiency than somewhat shorter pulses (see discussion on efficiency below, as well as Supplementary Section II.C of the Supplemental Information^27^).

Note that the Raman theory and simulations here have all been using cold plasma; equivalent results for warm plasma are discussed in detail in the Supplementary Information^27^. For plasma temperatures up to 100 eV and pump pulse intensities up to $4\times 10^{14}$ W/cm$^2$, we found no qualitative change to the results, and only a minor quantitative correction to Eq. (4).

### Efficiency of the amplification process

From the model by Malkin et al.^6^, we can already see that the first-peak efficiency in Raman amplification depends on the integrated amplitude $\epsilon$ of the initial seed pulse via $\eta = 4.4/\xi _M$, $\xi _M \approx 2.31 + \ln (1/\epsilon )$. Thus, the weaker the inital seed pulse, the smaller $\eta$, see the Supplementary Information^27^ for details. We also note that within the finite interaction distance of an experiment, only the first peak of the seed pulse may form fully, so the first-peak efficiency largely determines the overall efficiency. In this section, we will investigate the influence of the initial seed pulse strength on the efficiency via numerical simulations.

**Figure 3:**
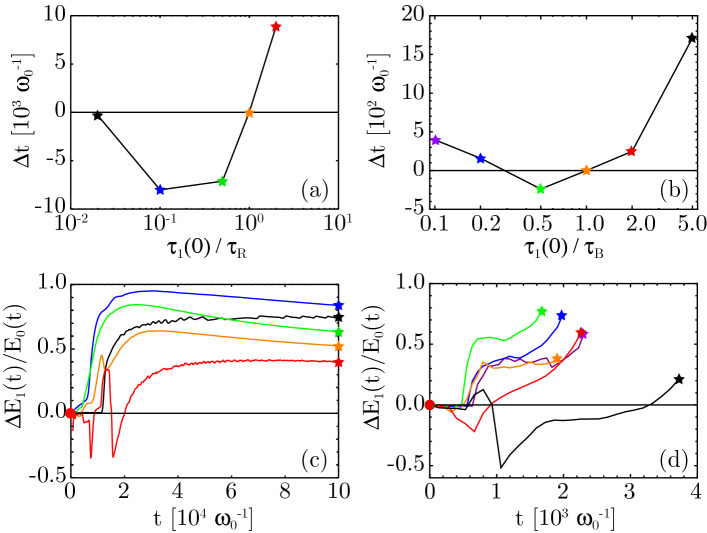
(**a**,**b**) Temporal delay $\Delta t$ in reaching a given intensity amplification level $(25\times I_\text {pump})$ for seed pulses with various initial durations, for the same cold-plasma cases as shown in frames (b,d) of Fig. 2. (**c**,**d**) efficiency of the amplification process for these same cases; $\Delta E_1$ is the seed energy gain, $E_0$ is the absorbed pump energy.

In Fig. 3a,b, we show the time (delay) needed for seed pulses with different initial durations to reach a given intensity $(25\times I_\text {pump})$, compared to a pulse with $\tau _1(0) = \tau _{R,B}$, for the same cases as shown in frames (b,d) of Fig. 2. A negative delay means that the given intensity is reached sooner than for the case with $\tau _1(0) = \tau _{R,B}$. We find that amplification is optimal when $0.2< \tau _1(0)/\tau _{R,B} < 0.5$, ($\tau _{R,B}$ given by (4) or (7)) while significant delays are incurred for $\tau _1(0)/\tau _{R,B} > 1$ or $< 0.2$. For an explanation why the fastest amplification and highest efficiency are not obtained for $\tau _1(0)/\tau _{R,B} = 1$, see the discussion on “transient” versus “asymptotic” solutions in Suppelementary Section II.C of the Suppelementary Information^27^. In Fig. 3c,d we show the efficiency of the amplification process for these same cases ($\Delta E_1 \equiv E_1(t) - E_1(0)$, so $\Delta E_1 < 0$ means that the seed pulse is losing energy rather than gaining). We find that (i) for Raman, the asymptotic efficiency is mostly constant, as predicted by Malkin et al.^6^ and above; (ii) for both Raman and sc-Brillouin, weak seed pulses lead to poor efficiency, while “optimal” seed pulses provide the best efficiency; (iii) if the initial seed pulse is “too long” for its amplitude, its duration will initially shrink, causing energy to flow back into the pump, resulting in “negative” efficiency, as also seen in results by Marquès et al.^18^; (iv) for both Raman and sc-Brillouin, the cases showing the longest delay also exhibit the lowest asymptotic efficiency. This means that a choice of seed pulse far from Eqs. (4) or (7) will harm the overall efficiency of the entire amplification process, not just that of the early stages. Also, the longest delays correspond to an interaction length of several mm, longer than what is used in many experiments^13,14,19,20,51^. The only solution here is to minimize the delay by using a seed pulse optimized according to our new non-linear matching conditions.

## Conclusions

We have explored the full non-linear evolution of the seed pulse in Raman and Brillouin amplification, and derived essential non-linear criteria for optimal amplification, namely that the triple product of the coupling constant $\Gamma$, the seed pulse amplitude $a_1$ (or $a_1^{2/3}$ for strongly coupled Brillouin amplification) and the seed pulse duration $\tau _1$ remains constant during amplification and is independent of the pump pulse properties. We have demonstrated the validity of these novel criteria in 1-D and 2-D particle-in-cell simulations.

Furthermore, we have demonstrated the importance of choosing the initial seed duration and amplitude wisely: non-optimal values for these parameters [far from the conditions (4) and (7)] will delay amplification of the seed pulse and reduce efficiency. We have compared the initial seed pulses used in all relevant Raman and Brillouin amplification experiments, and found that nearly all fall well short of our new criteria for efficient amplification. This goes a long way towards explaining why only the Brillouin amplification experiment by Marquès et al.^18^ demonstrated significant ($>10$%) energy transfer from the pump to a compact amplified seed pulse. In this experiment, amplification was either poor, good or “negative” when the initial seed pulse energy was below, at or above the value predicted by our model. Thus, we stress that in the first plasma-based parametric amplification experiment where a seed pulse is used that matches our new criteria, amplification to Joule level is found immediately^18^, proving how vital those criteria are to obtain efficient amplification. We also note that nonlinear amplification was probably achieved in two Raman experiments^13,20^, and that these experiments would benefit greatly from more powerful seed pulses obeying Eq. (4).

Since the ideal amplified seed pulse assumes the shape of a cnoidal wave^6,7,21^, our results also explain the “bursty behaviour” observed in Brillouin and Raman backscattering^52,53^, as the scattered radiation assumes a similar shape (a train of pulses). Since Eqs. (1)–(2) are very similar to those for Raman scattering in solid-state physics or non-linear optics (compare e.g. Refs.^21,22,54^ to Ref.^6^) our results will be useful for Raman and Brillouin scattering in general (not just in plasma), or to many optical three-wave process with Kerr or $\chi ^{(3)}$ non-linearity and counter-propagating pulses, ensuring a wide range of applications for our new criteria.

For future Raman or Brillouin amplification experiments, we have three recommendations: (i) use initial seed pulses for which the triple products $\Gamma _R \tau _1 a_1$ or $\Gamma _{sc} \tau _1 a_1^{2/3}$ match our novel nonlinear conditions (4) and (7); for most (but not all) experiments, this means that the initial seed pulse power and energy need to increase significantly; (ii) use the absolute output power or energy as a measure for success, instead of the “gain” relative to a tiny input pulse; (iii) provide complete envelope characterisation of the amplified pulse (as already provided by Refs.^12,13,18^), to demonstrate both amplification and pulse compression^12,30^, which proves that the amplification is indeed nonlinear.

## Methods

### Parameters of the numerical simulations

For the simulations in the main manuscript, we have used the particle-in-cell codes XOOPIC^49^ and OSIRIS^50^. The parameters are discussed in detail here. We distinguish numerical parameters (spatial resolution, time step, number of particles per grid cell) and physical parameters (laser pulse duration, spot diameter and amplitude, plasma density, plasma species, laser-plasma interaction length, etc.).

Both the Raman and Brillouin runs that have been performed for figures 1 and 2 of the main manuscript have been done using a moving simulation window. This window followed the seed pulse, while the pump pulse was brought into the simulation box via a time-dependent boundary condition on the leading edge of the moving window. Fresh plasma was loaded at the leading edge of the window for each time step, and the plasma particles were given a velocity consistent with the EM fields of the pump pulse.

The numerical parameters were as follows. For the Raman runs [frames (a) and (c) in both Figs. 2 and 3 of the main manuscript], the spatial resolution was 50 points per pump laser wavelength (i.e. $dx = 21$ nm). The time step was given by $dt = 0.95\cdot dx/c$. The number of particles was 100 particles per cell per species. The interpolation between particles and grid was done using quadratic splines. Ions were treated as an immobile background. For the Brillouin runs [frames (b) and (d) in both Figs. 2 and 3 of the main manuscript], the spatial resolution was $dx = 0.5\lambda _D$, where $\lambda _D$ is the Debye length. This corresponds to about 220 points per pump laser wavelength (i.e. $dx = 4.8$ nm). The time step was again $dt = 0.95\cdot dx/c$. The number of particles was again 100 particles per cell per species, and cubic splines were used for interpolation.

Boundary conditions are absorbing for fields and particles; only the injection of the pump beam deserves special attention. The pump beam is injected into the simulation box from the leading edge of the moving window, in the backward direction. To this end, a boundary condition $\mathbf {A}_\perp (\mathbf {x},t) = A_{0\perp } (\mathbf {x}_\perp ,t) \cos (-k_0 z - \omega _0 t)$ is applied to the fields at the leading-edge boundary. The variable *z* is the position of the leading edge, which changes every time the window is moved. When fresh plasma particles are injected at the leading edge, their transverse momentum is given by $\mathbf {p}_\perp = e\mathbf {A}_\perp$, to ensure that the canonical momentum $\mathbf {P} \equiv \mathbf {p} - e\mathbf {A}$ is conserved. This method was first implemented by P. Mardahl in 2001 for the code XOOPIC^49^; it has since been ported to the code Osiris^50^ also.

The physical parameters were as follows. For both Raman and Brillouin simulations, we used a long pump laser beam with constant amplitude $a_0$, linear polarisation ($g=1$) and wave length $\lambda _0 = 1\ \upmu$m ($\omega _0 = 2\pi c/\lambda _0$, $n_{cr} = \varepsilon _0 m_e \omega _0^2/e^2$). The seed laser pulse has initial amplitude $a_1(0) = a_0$, duration $\tau _1(0)$ (determined by the requirements of a particular configuration) and linear polarisation. The initial seed pulse envelope is given by $a_1(t,z) = ||a_1|| f((t-z/c)/\tau _1)$ with $f(x) = 1-[10-|x|(15-6|x|)]|x|^3$ for $|x| < 1$, zero otherwise. The seed laser wave length for the Raman simulations was 1.07 $\upmu$m, chosen to ensure that $\omega _1 = \omega _0 - \omega _{pe}$. The seed laser wave length for the Brillouin runs was 1 $\upmu$m. (This hardly matters since the frequency difference between pump and seed pulses in Brillouin amplification is considerably less than the seed pulse bandwidth.).

We used a long plasma column with constant electron density $n_0$ and plasma frequency $\omega _{pe}$ (see Refs.^47,55–57^ for a discussion on non-constant plasma densities). For the cold-plasma Raman simulations, we used a plasma density corresponding to $\omega _{pe}/\omega _0 = 1/15$, pump laser amplitudes $a_{wb}/4$, $a_{wb}/2$, $3a_{wb}/4$, $a_{wb}$ and $2a_{wb}$ where $a_{wb} \equiv \alpha _R/\sqrt{2} = 0.006086$, and pump pulse durations up to $2\times 10^5 /\omega _0 \approx 112$ picoseconds. We use $\tau _1(0)/\tau _R = 0.1$, 0.5, 1.0 and 2.0, where $\tau _R [\text {s}] = 4.22\times 10^{-6} \lambda _0 [\upmu \text {m}] (n_e/n_{cr})^{-1/4} (I_1 \lambda _1^2 [\text {W\;cm}^{-2} \upmu \text {m}^2])^{-1/2}$ is taken from (4). To test the influence of plasma temperature, selected cold-plasma runs were repeated at electron temperatures of $k_B T_e = 12$, 50 and 100 eV (see the Supplementary Information^27^ for details). For the Brillouin simulations, we used $m_i/(Zm_e) = 1836$, a plasma density $n_e = 0.3 n_{cr}$ and pump amplitudes $a_0= 0.0085$, 0.027 and 0.085, corresponding to $10^{14}$, $10^{15}$ and $10^{16}$ W cm$^{-2}$, and pump pulse durations of 11.4 ps, 3.8 ps and 1.1 ps, respectively. We use $\tau _1(0)/\tau _B = 0.1$, 0.2, 0.5, 1.0, 2.0 and 5.0, where $\tau _B [\text {s}] = 1.78\times 10^{-9} \lambda _0 [\mu \text {m}] [(Zm_e/m_i) (n_e/n_{cr}) (1-n_e/n_{cr}) (I_1 \lambda _1^2 [\text {W cm}^{-2} \mu \text {m}^2])]^{-1/3}$ is taken from Ref. (7).

The interaction distance for the simulations displayed in Fig. 3 is up to $10^5 c/\omega _0$ for the Raman runs, and up to $2\times 10^3 c/\omega _0$ for the Brillouin runs. For the Brillouin runs in Fig. 2, frame (b), the interaction length was about $2\times 10^4 c/\omega _0$, $8\times 10^3 c/\omega _0$, and $2\times 10^3 c/\omega _0$ for the simulations with pump intensity $10^{14}$, $10^{15}$ and $10^{16}$ W/cm$^2$, respectively. The interaction lengths for the simulations in Fig. 2, frame (d), are $2\times 10^3 c/\omega _0$ in each case. This corresponds to an interaction distance of 335 micron, or a 2.2 ps pump pulse duration. The interaction distance for the Raman runs in Fig. 2, frames (a) and (c), was up to $10^5 c/\omega _0$ or up to 16 mm in each case.

## Supplementary information

Supplementary Information.
