# Supplementary material for: New criteria for efficient Raman and Brillouin amplification of laser beams in plasma
Source: Sci Rep. 2020 Nov 16;10:19875. doi: 10.1038/s41598-020-76801-z (PMC7670465; doi:10.1038/s41598-020-76801-z)
Supplement: Supplementary file 1 — Supplementary Information. [file 41598_2020_76801_MOESM1_ESM.pdf]

Supplementary information to "New criteria for efficient Raman and Brillouin amplification of laser beams in plasma" by R. Trines *et al.*

R. M. G. M. Trines, E. P. Alves, E. Webb, J. Vieira, F. Fiùza, R. A. Fonseca, L. O. Silva, R. A. Cairns and R. Bingham

I. ANALYSIS OF EXPERIMENTS USED IN FIGURE 1 IN THE MAIN MANUSCRIPT

A. Raman amplification

The essential data for the Raman amplification experiments displayed in Figure 1 of the main manuscript are given in table I:

Expt.	ω_0 (rad/s)	n_0/n_{cr}	Γ_R (s^{-1})	τ_1 (s)	$\Gamma_R\tau_1$	a_1	$\Gamma_R\tau_1 a_1$	$\mathcal{E}_{\rm in}$ (mJ)	$\mathcal{E}_{\rm out}$ (mJ)	Eff. (%)
P1 [1]	3.54e15	0.0814	9.454e14	2e-13	189.1	1.56e-3	0.295	150	0.24	0.16
P2 [2]	2.346e15	0.007	3.386e14	5e-13	169.3	8.38e-4	0.1418	20	0.38(?)	1.9(?)
P3 [3]	2.346e15	0.0064	3.313e14	5.5e-13	182.2	1.59e-3	0.291	40	0.5	1.25
P4 [4]	2.346e15	0.0080	3.51e14	2e-13	70.3	1.53e-3	0.107	300	12	4
P5 [5]	2.346e15	0.0086	3.57e14	2e-13	71.5	1.12e-3	0.0798	250	1.4	0.56
R1 [6]	2.346e15	0.0075	3.425e14	5e-13	171.2	7.22e-4	0.1236			
R2 [6]	2.346e15	0.0075	3.425e14	9e-14	30.8	0.104	3.2	90	3.5	4
R3 [6]	2.346e15	0.0075	3.425e14	5.5e-14	18.8	0.165	3.1	90	5.6	6.2
L1 [7]	1.787e15	0.015	3.117e14	1e-12	311.7	7.61e-4	0.237	17.5e3†	6.7	0.1
L2 [8]	1.787e15	0.015	3.117e14	5e-12	1559	2.63e-4	0.4096	11e3†	14	0.32
L3 [9]	1.787e15	0.012	2.957e14	3.5e-12	1035	8.1e-4	0.83	11.5e3†	14	0.30
S1 [10]	2.346e15	0.0004	1.659e14	6e-13	99.5	9.8e-3	0.9754	220	7.7	3.5
S2 [11]	2.346e15	0.0004	1.659e14	6e-13	99.5	4.9e-3	0.4877	350	3.5	1
S3 [12]	2.346e15	0.0013	2.206e14	1.4e-13	30.88	0.0142	0.44	880	12	1.4
M1 [13]	2.346e15	2.5e-5	8.294e13	8e-14	6.6352	7.9e-4	5.24e-3	18	0.012	0.07
M2 [14]	2.346e15	0.002	2.481e14	8e-14	19.84	7.73e-3	0.1534	200	1	0.5
M3 [15]	2.346e15	0.004	2.95e14	3.8e-14	11.2	2.96e-3	0.033	345	0.73	0.2
M4 [15]	2.346e15	0.004	2.95e14	3.8e-13	112	9.36e-4	0.105	345	0.73	0.2

Table I: Data taken from Raman amplification experiments at Princeton University (P and R), Lawrence Livermore National Laboratory (L), the University of Strathclyde (S) and miscellaneous experiments (M). These data have been used to create Figure 1(a) of the main manuscript.

Here, ω_0 denotes the pump laser angular frequency, n_{cr} the critical density for that frequency, n_0 the plasma electron density, $\Gamma_R = \omega_0(n_e/n_{cr})^{1/4}/\sqrt{4g}$ is the Raman backscattering coupling coefficient, $g = 1$ ($g = 1/2$) denotes linear (circular) polarisation, τ_1 and a_1 are the signal pulse's duration and amplitude. In table I, P1-P5 denote experiments at Princeton University, L1-L3 denote experiments at Lawrence Livermore National Laboratory, S1-S3 denote experiments at Strathclyde University (Glasgow, United Kingdom) and M1-M4 denote miscellaneous experiments. R1-R3 denote pulses from the double-pass experiment by Ren *et al.* [6] at Princeton University: R1 is the signal pulse before interaction, R2 is the pulse after the first pass and R3 is the pulse after the second pass. We note that this is the only Raman amplification experiment that reported enough information on the parameters of the amplified pulses (both intensity and duration) to do our analysis. In particular, duration and envelope characterisation for the output pulse are missing from all other experiments.

Pulse parameters were determined as follows. Most papers provide the energy, duration, wave length and spot diameter of the initial signal pulse and the background plasma density in an unambiguous fashion. The polarisation of the laser beams is not always given, but can usually be taken as linear, especially when Ti:sapphire lasers are used [16]. Energy and duration yield power, power and spot diameter yield intensity, intensity and wave length yield a_1. The pump pulse wave length yields ω_0 and n_{cr}, and together with the plasma density this yields Γ_R. Thus, we find all factors of the triple product $\Gamma_R\tau_1 a_1$. This was fairly straightforward for the initial signal pulses of almost all experiments. However, in the 2011 experiment by Kirkwood *et al.* [9], a thermal plasma was assumed with a

dispersion relation $(\Delta\omega/\omega_0)^2 = n_e/n_{cr} + 12k_BT_e/(m_ec^2)$, where $\Delta\omega$ denotes the difference between pump and signal pulse frequencies. Then $(\Delta\omega/\omega_0)^2 = (1 - 1054/1220)^2 = 0.0185$ was found, and $k_BT_e \sim 250$ eV was taken from a simulation to infer $n_e/n_{cr} \sim 0.012$. Neither n_e nor T_e were measured directly though.

Regarding pulse energies: $\mathcal{E}_{\rm in}$ is the energy of the input pump beam, while $\mathcal{E}_{\rm out}$ is the energy of the output (amplified) seed pulse, both as listed in the publication. No output energy is given for pulse R1, since this is a double-pass experiment with two output energies, after one (R2) or two (R3) passes. The highest efficiencies have been obtained by experiments P4 and the Ren experiment, both conducted at Princeton [4, 6]. This is mainly due to the somewhat larger spot diameters that could be maintained in these experiments (see below). For the Livermore experiments L1-L3, we find that the pump pulse is 1 nanosecond long, while the plasma column is only 3 mm wide, so only 20 picosecond (2%) of the pump beam can interact meaningfully with the seed pulse. To improve the comparison with other experiments, we have used 2% of the published pump pulse energy in table I for these experiments (marked with †). In spite of this, we find that the efficiencies of the Livermore experiments remain below 1%, below those reported by Princeton or Strathclyde. In fact, the level of Raman backscattering in the Livermore experiments is suspiciously low [7–9], as evidenced by the null shot (only pump, no seed) in Figure 5 of Ref. [8]. In this shot, a 220 J pump beam (1 ns duration and 0.2 mm spot diameter, so 0.22 TW and 5.5×10^{14} W/cm^2, $a_{00} = 0.016$) is launched at a 3 mm plasma column with $\omega_0/\omega_p = 8$ (i.e. quite high density, $\Gamma_R = 3.1 \times 10^{14}$ s^{-1}). Even so, hardly any Raman backscattering is seen here, although $\Gamma_R a_{00} L/c \sim 50$ for this shot. Thus, it seems that the Raman backscattering instability itself is inhibited in the Livermore experiments. This may have been caused by their use of random phase plates to smooth the pump beam. Random phase plates are best known as devices to smooth laser beam envelopes [19]. However, they are also known to suppress laser-plasma instabilities like stimulated Raman and Brillouin backscattering because they reduce the spatial coherence of the laser light [20–24], which may be bad for Raman amplification. Princeton and Strathclyde do not report the use of random phase plates, while the LULI Brillouin experiments only use them for the ionizing beam, not for the pump and seed beams. The reduction of the growth rates for stimulated Raman scattering due to an increase in wave number bandwidth (and thus a reduction of the spatial coherence) has also been studied by Santos *et al.* [25].

It is of interest to study the correlation between the value of the triple product $a_1\Gamma_R\tau_1$ (or $a_1^2\Gamma_{sc}^3\tau_1^3$ for sc-Brillouin) for the initial seed pulses, and the efficiency obtained in the experiments using those seed pulses. For Raman amplification, a weak positive correlation is found between the values for efficiency and the triple product: $R = 0.16$. The small value for R has two causes: (i) there are no Raman experiments with large values for either the triple product or the efficiency, so it is difficult to spot any trend, and (ii) the Princeton experiments P4 [4] and Ren [6] report much higher efficiencies ($\sim$ 4%) than one would expect from their values for the triple product ($\sim$ 0.1). Without those two experiments, the correlation between triple product and efficiency would be much stronger: $R = 0.58$. For sc-Brillouin amplification, we find a strong positive correlation: $R = 0.62$. This is mainly driven by the results of the Marquès experiment [29], which covers wide ranges of values for both the triple product $a_1^2\Gamma_{sc}^3\tau_1^3$ and the efficiency, with a strong correlation between them.

From Figure 1 in the main manuscript, certain trends can be observed: experiments at Livermore National Laboratory use high plasma densities, long pulse durations and low amplitudes, while experiments at Strathclyde University use low densities and short pulse durations, but higher amplitudes. Experiments at Princeton University occupy the middle ground. We note that none of the input pulses satisfies our new criterion $\Gamma_R\tau_1a_1 \approx 3.4$, usually falling far short. However, if $\Gamma_R\tau_1$ from one of the Livermore experiments could be combined with a_1 from one of the Strathclyde experiments, a seed pulse with a good value for $\Gamma_R\tau_1a_1$ would result.

In many Raman amplification experiments with weak seed pulses, the growing seed pulse may only reach the nonlinear regime towards the end of the interaction. This can be explained as follows. In most experiments, the initial seed pulse has a finite duration in the range of 0.1-1 ps. When its amplitude is too low, the first effect we observe is not seed pulse growth, but seed pulse stretching, see e.g. Section III of the main manuscript. From figure 3 in that section, we find that this process typically takes about 1000 laser cycles, or 1 mm for a pump laser with 1 micron wave length. The experiments carried out at Princeton and Strathclyde use plasma columns of about 1 mm in length, so nearly the entire plasma column is used up for laser pulse stretching rather than amplification. This goes some way towards explaining the poor efficiency of those experiments. A second cause of inefficiency in this experiment is the reduction of the seed pulse spot diameter, see Section I C below.

B. Brillouin amplification

The essential data for the Brillouin amplification experiments displayed in Figure 1(b) of the main manuscript are given in table II:

Quantities have the same meaning as before; $\Gamma_{sc}^3 = \omega_0^3(Zm_e/m_i)(n_0/n_{cr})(1-n_0/n_{cr})/(2g)$ is the coupling coefficient for strongly-coupled Brillouin scattering, where Zm_e/m_i is the electron-ion charge-mass ratio. L1 and L2 denote the

Expt.	ω_0 (rad/s)	n_0/n_{cr}	Γ_{sc} (s^{-1})	τ_1 (s)	$\Gamma_{sc}\tau_1$	a_1	$a_1^2\Gamma_{sc}^3\tau_1^3$	$\mathcal{E}_{\rm in}$ (J)	$\mathcal{E}_{\rm out}$ (J)	Eff. (%)
L1 [26]	1.787e15	0.1	2.410e13	4e-13	9.64	0.0637	3.63	2	0.06	3
L2 [27]	1.787e15	0.1	5.191e13	7e-13	36.34	5.26e-3	1.33	6	0.03	0.5
G1 [28]	1.787e15	0.17	4.786e13	1e-12	47.86	0.016	28.4	0.68	0.017	2.5
Mi1 [29]	1.780e15	0.05	4.178e13	5.5e-13	22.98	9.36e-3	1.065	9	0.45	5
Mi2 [29]	1.780e15	0.05	4.178e13	5.5e-13	22.98	0.0296	10.65	9	0.85	9.5
Mi3 [29]	1.780e15	0.05	4.178e13	5.5e-13	22.98	0.0662	53.25	9	1.75	19.5
Mi4 [29]	1.780e15	0.05	4.178e13	5.5e-13	22.98	0.296	1065	9	-2.05	-23
Mo1 [29]	1.780e15	0.05	4.178e13	2.9e-13	12.09	0.162	46.3	9	0.85	9.5
Mo2 [29]	1.780e15	0.05	4.178e13	1.44e-13	6.0	0.229	11.6	9	0.85	9.5

Table II: Data taken from Brillouin amplification experiments at the Laboratoire d'Utilisation des Lasers Intenses (LULI), France (L), the experiment by Guillaume *et al.* at the Rutherford Appleton Laboratory (G), and the recent experimen by Marquès *et al.* at LULI (Mi and Mo). These data have been used to create Figure 1(b) of the main manuscript.

input pulses for the first two experiments at LULI (Palaiseau, France) by Lancia *et al.* [26, 27], and G1 denotes the input pulse for the experiment at the Central Laser Facility (RAL, Didcot, United Kingdom) by Guillaume *et al.* [28]. Mi1-Mi4 denote input pulses from the recent experiment by Marqués *et al.* [29], having energies of 4, 40 and 200 mJ and 4 J respectively. We note that the best results were obtained for pulses Mi2-Mi3, which match our criteria best (an initial seed energy of 52 mJ would have been optimal for this experiment), while results were not as good for Mi1 and Mi4, which do not match our criteria. We also note that the points in Figure 3a of Ref. [29] form an S-curve, whose centre sits roughly at 40-50 mJ, just where our criteria predict that it should be.

Obtaining the duration of the output pulses Mo1 and Mo2 was more involved, since those durations are not stated explicitly in Ref. [29]. The procedure was as follows. We noted that the duration of the seed pulse in vacuum was given as 0.55 ps, while the FWHM of the autocorrelator trace of this same pulse (black curve in Fig. 5a) is found to be 0.95 ps. From this, a deconvolution factor of 1.73 is estimated. We then take the FWHM of the central peak of the purple curve in Fig. 5b, 0.5 ps, and apply the deconvolution factor to obtain an estimated pulse duration of 0.29 ps. Together with the pulse energy of ~ 1 J and a spot diameter of 0.1 mm, this yields point Mo1. However, as explained below under "Transverse effects" and clearly shown in Fig. 8 of Ref. [29], the amplified pulse takes on a "horseshoe" shape, which can easily lead to a factor 2 overestimation of its duration in the autocorrelator trace. Applying this correction to τ_1 increases a_1^2 by a factor 2, while decreasing $a_1^2\Gamma_R^3\tau_1^3$ by a factor 4. This leads to point Mo2 in table II, which matches our criterion better than Mo1. Obviously, there are many uncertainties in our approach, caused by the paucity of the available data. In order to do a proper analysis of the output pulses, more accurate characterisation of these pulses is needed.

Regarding pulse energies: again, $\mathcal{E}_{\rm in}$ and $\mathcal{E}_{\rm out}$ denote the energies of the input pump and output (amplified) seed pulse as published. The highest efficiencies have been obtained by pulses Mi2 and Mi3, whose values of $a_1^2\Gamma_{sc}^3\tau_1^3$ are closest to the predictions by our model. Pulse Mo4 is so far above the predicted value initially that energy flows back from the seed pulse into the pump pulse; therefore this pulse has a "negative" efficiency. Experiment G1 has a seed pulse that matches our criteria, but the interaction length is too short for the given pump intensity, so this experiment did not reach its full potential. To compare: Guillaume *et al.* [28] use $I_{\rm pump} = 3\cdot 10^{14}$ W/cm^2, $\tau_{\rm pump} = 15$ ps, and 1.65 mm interaction length (which is too short for the given pump duration), while Marquès *et al.* [29] use $I_{\rm pump} = 4\cdot 10^{16}$ W/cm^2, $\tau_{\rm pump} = 1.7$ ps, and 1.2 mm interaction length. If the Guillaume experiment would be repeated with a longer interaction length (e.g. 3-4 mm) and a somewhat higher pump intensity (e.g. $I_{\rm pump} \sim 10^{15}$ W/cm^2), significantly higher efficiencies could probably be obtained.

C. Output pulse analysis

In contrast to the input pulses, the output pulses of several experiments [3, 4, 6, 17, 18, 26] show promise. In those experiments where a full characterisation of the output pulse is actually provided, this is usually given in terms of the pulse's energy, duration and spot diameter. The relationships between those parameters and the "triple products" $a_1\Gamma_R\tau_1$ or $a_1^{2/3}\Gamma_{sc}\tau_1$ given in Eqns. (7) and (13) below are as follows:

$$(\Gamma_R\tau_1 a_1)^2 = (4/\pi)(8.55\times 10^{-6}\times \Gamma_R\sqrt{g}\lambda_1/R_1)^2\mathcal{E}_1\tau_1, \tag{1}$$

$$a_1^2(\Gamma_{sc}\tau_1)^3 = (4/\pi)(8.55\times 10^{-6}\times \sqrt{g}\lambda_1/R_1)^2\Gamma_{sc}^3\mathcal{E}_1\tau_1^2. \tag{2}$$

Here, $\mathcal{E}_1$ is the energy of the amplified seed pulse in Joule, and τ_1 its duration in seconds, while λ_1 and R_1 are the seed pulse's wave length and spot diameter. We apply these equations to the output pulses discussed in various papers. For Ren *et al* [6], we find $a_1\Gamma_R\tau_1 = 3.66$ after the first pass, and $a_1\Gamma_R\tau_1 = 4.05$ after the second pass, close to the theoretical prediction of $\Gamma_R\tau_1 a_1 \sim 3.4$ above, as already discussed above. For experiment P4 by Turnbull *et al.* [4] we find an output pulse with 12 mJ energy and $\tau_1 \sim 0.44$ ps duration for a plasma with $\omega_{pe}/\omega_0 \sim 0.1$. Using the pump pulse diameter of 0.1 mm (the output pulse's spot diameter was not given), we find $I_1 \sim 2.7 \times 10^{14}$ W/cm^2, $a_1 \sim 0.01$ and $\Gamma_R\tau_1 a_1 \sim 2$, (or $\Gamma_R\tau_1 a_1 \sim 4$ if a 50 μm spot diameter is assumed). For the Brillouin amplification experiment L1 [26], we find $\mathcal{E}_1 \sim 60$ mJ, $\tau_1 \sim 0.2$ ps, $R_1 \sim 17$ μm, which yields $I_1 \sim 2 \times 10^{17}$ W/cm^2, $a_1 = 0.28$ and $a_1^2(\Gamma_{sc}\tau_1)^3 \sim 9.2$, not too far from the predicted value of 13.8. We find that in all those experiments, the amplified seed pulse is at or near the non-linear regime at least within its central spot, where its intensity is highest.

Furthermore, we note that experiment P3 [3] includes a graph (figure 5 of Ref. [3]) of the duration τ_1 versus the energy $\mathcal{E}_1$ of the amplified seed pulse. The same is true for later papers on the Ren experiment [17, 18]. We use equation (1) to analyze this data. For Ref. [3] we use $\lambda_1 = 873$ nm and $R_1 \sim 8$ μm, while for Refs. [17, 18] we use $\lambda_1 = 873$ nm and $R_1 \sim 15$ μm. The main uncertainties in this approach are related to the measurements of the seed pulse energy and spot diameter. We note that curve fitting with $\tau_1 = \alpha\mathcal{E}_1^{-\beta}$ does not work here (see below). Instead, one should simply use the value of the triple product $\Gamma_R\tau_1 a_1$ to judge how well the output pulse resembles a perfect π-pulse.

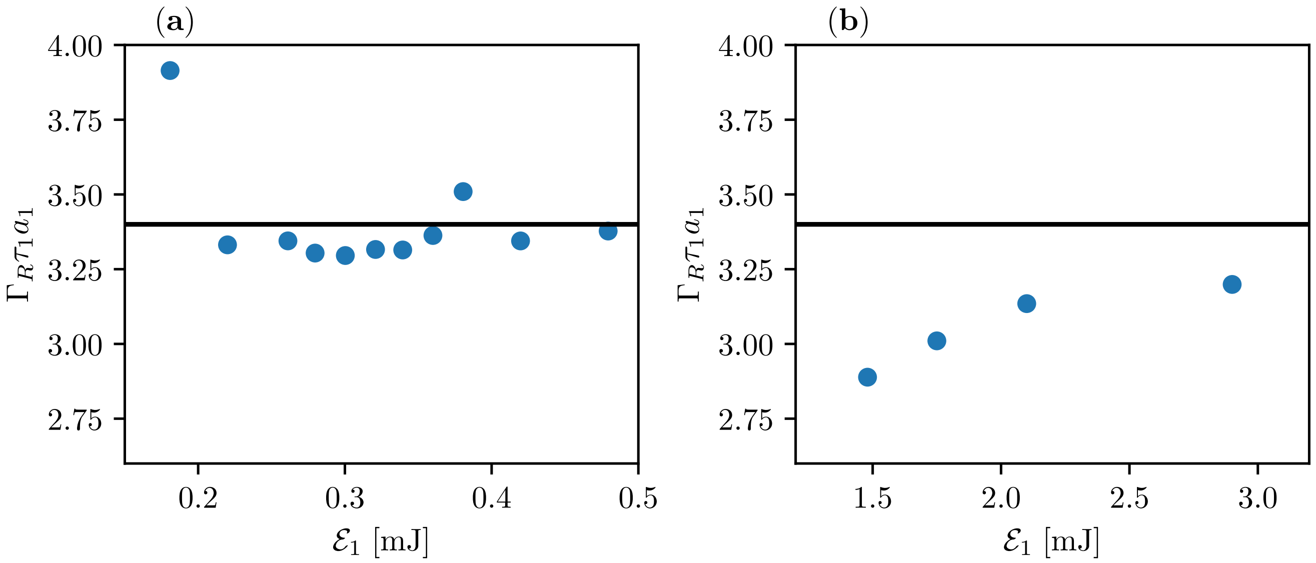

Figure 1: Analysis of the $(\tau_1, \mathcal{E}_1)$ data from experiment P3 [3] (a) and the Ren experiment [17, 18] (b). Shown is the value of $\Gamma_R\tau_1 a_1$ versus $\mathcal{E}_1$ [mJ]: blue dots indicate the experimental data, the black line the theoretical prediction. The experimental error in $\Gamma_R\tau_1 a_1$ is about 20% in frame (a), 10% in frame (b). We note that the value of $\Gamma_R\tau_1 a_1$ for experiment P3 is nearly constant, and close to the theoretical value 3.4, while the values of $\Gamma_R\tau_1 a_1$ for the Ren experiment are approaching 3.4 asymptotically. This means that the seed pulses of experiment P3 are more non-linear inside their spot than those of the Ren experiment, in spite of the higher energies obtained in the Ren experiment.

We harvested the data from Refs. [3, 17, 18] and analyzed it; the results are displayed in Figure 1. While both experiments show clear signatures of proper non-linear Raman amplification, we find that the value of $\Gamma_R\tau_1 a_1$ for experiment P3 is nearly constant (see Figure 1(a)), and close to the theoretical value 3.4, while the values of $\Gamma_R\tau_1 a_1$ for the Ren experiment are approaching 3.4 asymptotically (see Figure 1(b)). This means that the seed pulses of experiment P3 are more non-linear inside their spot than those of the Ren experiment, in spite of the higher energies obtained by Ren *et al.* Furthermore, we note that all the output pulses for which we could do an energy analysis are mostly or fully non-linear. Thus, the success of a Raman or Brillouin amplification experiment is no longer determined by its degree of non-linearity. Other parameters are becoming more important, in particular the spot diameter of the amplified seed pulse. This is expressed in table III:

In table III, R_0, R_{1i} and R_{1f} denote the spot diameters of the pump pulse, initial seed pulse and final seed pulse respectively; τ_{1f}, a_{1f} and "tr. prod." denote the duration, amplitude and triple product ($\Gamma_R\tau_1 a_1$ or $a_1^2(\Gamma_{sc}\tau_1)^3$ as appropriate) for the seed pulse after amplification.

Expt.	$\mathcal{E}_{\rm out}$ (mJ)	$\tau_{\rm 1f}$ (fs)	R_0 (μm)	$R_{\rm 1i}$ (μm)	$R_{\rm 1f}$ (μm)	$a_{\rm 1f}$	Tr. prod.	Eff. (%)
P3 [3]	0.48	170	55	55	8	0.06	3.38	1.25
Ren [17, 18]	2.9	90	55	55	15	0.104	3.2	3.2
R3 [6]	5.6	55	55	55	15	0.165	3.1	6.2
P4 [4]	12	440	100	100	50-100 (?)	0.01-0.02	2-4	4
L1 [26]	60	200	30	20	17	0.28	9.2	3
Mo2 [29]	1.2e3	250	100	80	~100	0.229	11.6	9.5

Table III: Output pulse data for selected Raman and Brillouin amplification experiments, for which sufficient data had been provided in the corresponding publications. Shown are data from two Raman experiments at Princeton University (P3 and P4), from the experiment by Ren *et al.* [6] after the first pass (Ren) and after the second pass (R3), and from two Brillouin experiments at LULI (L1 and Mo2).

From the data in table III, we find that the experimets P3, Ren and L1 [3, 6, 26] suffer from a significant spot size reduction during amplification. This is the main reason why the output pulses in those experiments carry so little energy, even though they appear to have reached the non-linear regime within their spots. This reduction in spot diameter is likely caused by the initial seed pulse being so weak that it does not have enough intensity in its wings to kick-start non-linear amplification. Non-linear amplification then takes place only within a narrow spot near the axis of propagation, while there is no amplification at all away from the axis. Furthermore, we see that the Ren experiment [6] produced a more energetic output pulse and received wider publicity mainly because of its larger spot diameter, not because of its degree of non-linearity. Experiment P4 reports an output seed energy of 12 mJ, one of the highest ever reported in a plasma-based Raman amplification experiment. This result is mainly due to its fairly large spot diameter (50-100 μm); the long duration and low amplitude of the output seed pulse indicate that the amplification had not yet reached an advanced stage. The Brillouin amplification experiments L1 [26] and Marquès [29] have comparable values for a_1, τ_1 and $a_1^2(\Gamma_{sc}\tau_1)^3$, but the output pulses by Marquès *et al.* contain much more energy because amplification could be maintained across a much wider spot diameter (100 μm vs. 17 μm). Each time, we see that amplification across a larger spot diameter lies at the base of increased efficiency of the process. To compare: in a recent experiment on crossed-beam energy transfer (CBET, [30]), a large amount of energy could be transferred between laser beams interacting in plasma because the transfer took place across a large spot (300-600 μm).

Further analysis is also possible using Eq. (6) below. We calculate $a_0^2\Gamma_R^2\tau_0\tau_1$ for both the most energetic pulse from experiment P3 [3] ($a_0 = 0.01$, $\Gamma_R = 3.313 \times 10^{14}$ s^{-1}, $\tau_0 = 10$ ps, $\tau_1 = 150$ fs, $a_0^2\Gamma_R^2\tau_0\tau_1 \sim 16$) and for the pulse R2 after the first pass of the Ren experiment [6] ($a_0 = 0.01$, $\Gamma_R = 3.425 \times 10^{14}$ s^{-1}, $\tau_0 = 7$ ps, $\tau_1 = 90$ fs, $a_0^2\Gamma_R^2\tau_0\tau_1 \sim 7$). Given that the initial seed pulses are well below the threshold for non-linear amplification, these values seem to be on the low side, indicating that the seed pulse is shorter (and thus more intense) after the given interaction length than could be expected from Raman amplification alone. This points to self-focusing as a contributing factor to the intensity increase, in line with the measured spot diameter decrease. Since self-focusing does not contribute to the total seed pulse power but does lead to a reduction in seed pulse duration, it follows that it has a negative effect on the total seed pulse energy and the efficiency of the amplification process.

Thus, in order to produce seed pulses with high power and energy using Raman or Brillouin amplification, it is vital to ensure that the amplification process takes place across a large spot diameter. To this end, it is vital to use seed pulses that satisfy Eq. (7) or Eq. (13) from the start, and do so across a large spot. Preferrably, the initial seed pulse spot diameter should be *larger* than the pump pulse diameter, not smaller as is sometimes advised [26, 29].

D. Effect of the intensity of the input seed pulse on the amplifier output

In support of our findings, we have conducted 2-D particle-in-cell simulations using the code XOOPIC [55] where a 10^{14} W/cm^2 pump pulse interacted with a seed pulse in a 4 mm long plasma column, with $\omega_0/\omega_p = 20$ or 10. We decreased the seed pulse intensity from 10^{15} W/cm^2 to 10^{12} W/cm^2, leaving all other parameters constant, and also included "null" simulations with a pump beam but no seed pulse at all. Simulation results are displayed in Figure 2. We found the following. (i) For every factor 10 decrease in the input seed intensity, we found a factor $\sim \sqrt{10}$ decrease in the output seed intensity. If we assume that the amplified seed pulse is mostly non-linear on-axis, so $\Gamma a_1\tau_1$ does not vary much between simulations, the energy flux is proportional to $a_1^2\tau_1 \propto a_1$, and will thus be reduced by a factor $\sqrt[4]{10} \approx 1.78$. This confirms the need to keep the input seed intensity up to achieve a high output seed intensity and power over a fixed finite interaction length. (ii) The transverse pulse width decreases with decreasing

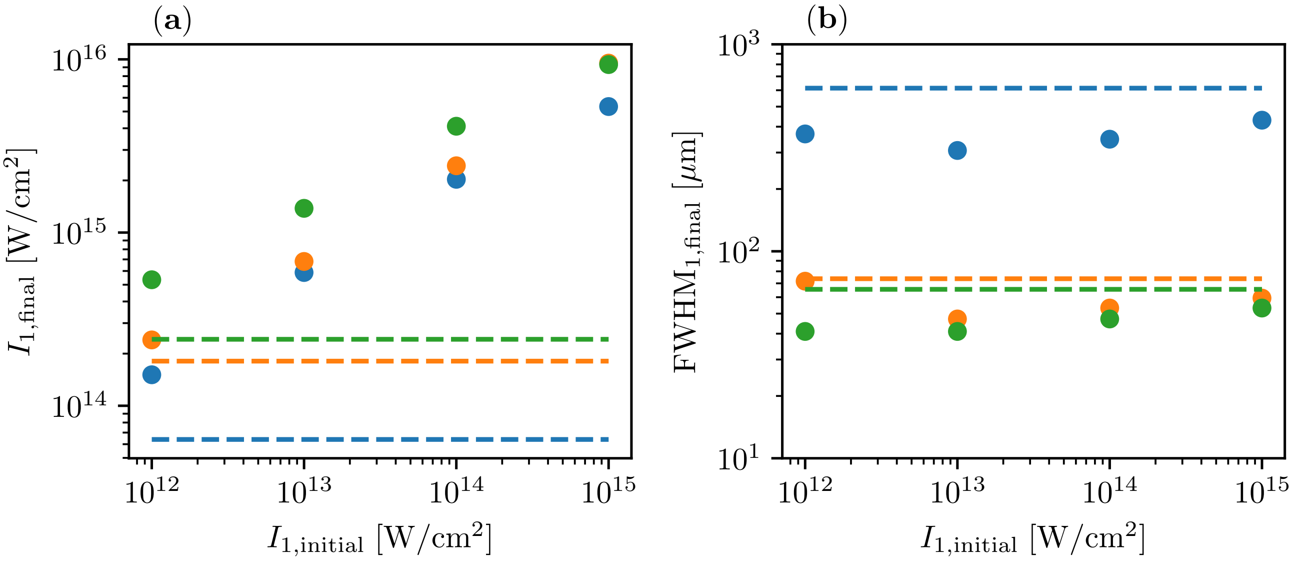

Figure 2: Results of two-dimensional XOOPIC simulations to study the result of a decrease in initial seed intensity when other parameters are kept constant. For all simulations, the pump intensity was 10^{14} W/cm^2 and interaction length 4 mm (i.e. $\sim$ 27 ps pump duration). Blue dots and line: initial seed pulse duration is 50 fs, pulse spot diameter is 600 micron, plasma electron density is $n_{cr}/400$. Orange: initial seed pulse duration is 200 fs, pulse spot diameter is 60 micron, plasma electron density is $n_{cr}/400$. Green: initial seed pulse duration is 50 fs, pulse spot diameter is 60 micron, plasma electron density is $n_{cr}/100$. (a): Final seed intensity versus initial seed intensity. The dashed line represents the level of pump Raman backscatter induced by noise in a simulation with no seed pulse. Each decrease of the initial seed pulse intensity by a factor 10 leads to a decrease in output intensity by a factor $\sim \sqrt{10}$. In the simulations with the lowest initial seed intensity, 10^{12} W/cm^2, the contribution of RBS from noise becomes comparable to the amplified seed. (b) Final seed spot FWHM versus initial seed intensity. The dashed line represents the FWHM of pump Raman backscatter induced by noise in a simulation with no seed pulse. For $I_{10} \geq 10^{13}$ W/cm^2, each decrease of the initial seed pulse intensity by a factor 10 leads to a decrease in final seed FWHM by 15-20%. In the simulations with the lowest initial seed intensity, 10^{12} W/cm^2, the contribution of RBS from noise causes the final spot FWHM to increase again.

initial seed intensity, by 15-20% for a factor 10 decrease of the input seed intensity, reaching a minimum for 10^{13} W/cm^2 initial seed intensity. (iii) Between the decrease in intensity and in spot diameter, we find that the efficiency of the process decreases by a factor $\sim$ 2.5 for a factor 10 decrease of the input seed intensity. (iv) Every time we decrease the input seed intensity, the relative importance of noise (and Raman-amplified noise) and instabilities like filamentation increases. For the lowest input seed intensity, 10^{12} W/cm^2, noise-driven RBS makes up about a third of the output signal. (v) Filamentation leads to the emergence of high intensity "speckles" on top of the laser envelope where the intensity gain is driven in part by the filamentation instability rather than Raman amplification (like in the experiment by Ren *et al.*). This does not lead to an overall increase amplified power though. (vi) For high initial seed pulse powers and intensities, the noise and noise-related instabilities are drowned out by the "regular" Raman amplification process.

We conclude that both the efficiency of the amplification and the quality of the pulse envelope decrease with decreasing pulse intensity, and speckles develop; while the speckles reach a relatively high intensity, the average output intensity and power decrease; efficient amplification to high powers across a wide spot with a good envelope is only seen for high input seed pulse intensities.

We repeated our simulations with a 10^{15} W/cm^2 pump pulse, leaving other parameters the same, but this led to a significant increase in noise and noise-driven instabilities, so we did not pursue this further.

Since our simulations without any seed pulse still showed significant Raman scattering from noise, we have investigated the importance of noise-driven Raman backward scattering (RBS) in experiments, wherever information was published on a "pump only" shot with no seed pulse. We looked at the energy from "thermal RBS" as a percentage of the energy of the amplified seed. Results were as follows. Princeton [1–6]: 10-30%, similar across experiments. Livermore (LLNL) [7–9]: 15-50% depending on the experiment. For Brillouin amplification, we find: LULI 2010 [26]: 10-15%. LULI 2016 [27]: 10%. LULI 2019 [29]: up to 25%, probably because of the large pump intensity (4×10^{16} W/cm^2). Clearly, the energy of the output pulse in all relevant experiments contains a sizeable contribution of RBS

from thermal noise, similar to what we see in our simulations. This cannot be fully eliminated, but its relative importance can be reduced by using a more powerful seed pulse and (for the LULI 2019 experiment) a reduction of the pump pulse intensity.

II. DERIVATION OF EQUATIONS (3), (4), (6) AND (7) IN THE MAIN MANUSCRIPT, AND THEIR PROPERTIES

We define a_0 and a_1 to be the scaled envelopes of pump and seed pulse respectively, $a_{0,1} \equiv 8.55 \times 10^{-10} g^{1/2}(I_{0,1}\lambda_{0,1}^2[\mathrm{Wcm^{-2}}\mu\mathrm{m}^2])^{1/2}$, where $g = 1$ ($g = 1/2$) denotes linear (circular) polarisation. Let ω_0, k_0 and n_{cr} denote the pump laser frequency, wave number and critical density, and n_e and ω_{pe} the background electron density and corresponding plasma frequency. The group velocity of the pump pulse is $v_g = c^2k_0/\omega_0 = c(1 - n_e/n_{cr})^{1/2}$ and the electron thermal velocity is $v_e = (k_BT_e/m_e)^{1/2}$. We note that we use $a_{0,1} = ||a_{0,1}|| \cos(\theta_{0,1})$ for the laser pulses, and $\delta n_e = ||\delta n_e|| \cos(\theta_L)$ for the Langmuir wave (where θ denotes a fast phase), same as in Forslund *et al.* [31] (and contrary to Drake *et al.*, who use $E = 2E_0 \cos(\theta)$), so the relationship between the amplitude of a real-valued physical quantity and the amplitude of its (sometimes complex) envelope function is obvious.

We will see below that the growth rates $\Gamma_{R,B}a_0$ and $\Gamma_{sc}a_0^{2/3}$, the wave-breaking conditions $a_0/\alpha_{R,B} > 1/\sqrt{2}$ and the threshold for a_0^2/g for strongly coupled Brillouin scattering all depend on a_0 and g only via the combination $a_0/\sqrt{g} = 8.55 \times 10^{-10}(I_0\lambda_0^2[\mathrm{Wcm^{-2}}\mu\mathrm{m}^2])^{1/2}$, which is independent of the laser polarisation g. The reason we include the polarisation explicitly is, that many theoretical works write these expressions in terms of the amplitude a_0 without being too clear about the effects of polarisation: Forslund *et al.* [31] and Malkin *et al.* [33] use circular polarisation, Andreev *et al.* [52] "probably" use circular polarisation, while Drake *et al.* [32], Kruer [34] and Lehmann and Spatschek [53] use linear polarisation. As a result, all works claim to provide "the" growth rate or "the" threshold, but they never seem to agree on the numerical coefficients in their expressions. Thus, we supply expressions with explicit polarization dependence to link theory papers with both linear and circular polarization to each other and to experiments. Our readers can then decide for themselves.

A. Raman amplification

For Raman amplification, the envelope equations for pump, seed and plasma wave can be written in the following generic form [31, 33]:

$$(\partial/\partial t + v_g\partial/\partial x)a_0 = -i\Gamma_R a_1 b, \tag{3}$$
$$(\partial/\partial t - v_g\partial/\partial x)a_1 = i\Gamma_R a_0 b^*, \tag{4}$$
$$(\partial/\partial t + 3v_e^2(k/\omega_{pe})\partial/\partial x)b = -i\Gamma_R a_0 a_1^*, \tag{5}$$

Here, a_0 and a_1 denote the scaled envelopes of pump and seed pulse respectively, $a_{0,1} \equiv 8.55 \times 10^{-10} \times g^{1/2}(I_{0,1}\lambda_{0,1}^2[\mathrm{Wcm^{-2}}\mu\mathrm{m}^2])^{1/2}$, where $g = 1$ ($g = 1/2$) denotes linear (circular) polarisation. Let ω_0, k_0 and n_{cr} denote the pump laser frequency, wave number and critical density, $v_e = (k_BT_e/m_e)^{1/2}$ the electron thermal velocity, and n_e and ω_{pe} the background electron density and corresponding plasma frequency. We define $b = \alpha_R\delta n_e/n_e$ where δn_e is the plasma wave density fluctuation and α_R is to be determined, and Γ_R is the Raman coupling constant, also to be determined (so the Raman backscattering growth rate in homogeneous plasma is given by $\Gamma_R a_0$). The group velocity of the pump pulse is then $v_g = c^2k_0/\omega_0 = c(1 - n_e/n_{cr})^{1/2}$.

Comparing these equations to the envelope equations (51) of Forslund *et al.* [31] yields $\Gamma_R\alpha_R = \omega_{pe}^2/(4\omega_0)$ and $\Gamma_R/\alpha_R = c^2k^2/(4g\omega_{pe})$, where $k \approx 2\omega_0/c$ is the wave number of the RBS Langmuir wave (for $\omega_{pe} \ll \omega_0$). Then $\Gamma_R = [\omega_0\omega_{pe}/(4g)]^{1/2}$ and $\alpha_R = \sqrt{g}(\omega_{pe}/\omega_0)^{3/2}/2$, with (3)-(5) valid for $a_0 < a_{wb} = \alpha_R/\sqrt{2}$ [33]. To derive the wave-breaking threshold for a_0, we use Eqns. (3) and (5) to derive the Manley-Rowe relation $2||a_0||^2 = ||b||^2$, and use the wave breaking condition $\delta n_e/n_e = 1$ [35], from which the wave breaking threshold $a_{wb} = \alpha_R/\sqrt{2}$ follows immediately.

Curiously, if the same reasoning is used to match the equations by Malkin *et al.* [33] to those by Forslund *et al.* [31], one finds $f = (\omega_{pe}/4\omega_0)(\delta n_e/n_e) = \bar{E}_L/2$ for the Langmuir wave (Ref. [33] uses f in lieu of our b), where $\bar{E}_L = eE_L/(m_e\omega_{pe}c)$ is that wave's scaled electrostatic field. This in spite of the fact that Malkin *et al.* state that "f is the electrostatic field of the Langmuir wave, normalized to $m_e\omega_{pe}c/e$", i.e. $f = \bar{E}_L$. Similarly, in papers describing the MBRS simulation code to solve the equations (3)-(5) [36, 37], it is claimed that "f is the complex amplitude of the Langmuir wave scalar potential in units $(mc^2/2e)\sqrt{\omega_p/2\omega_0}$", which would correspond to $f = 2\alpha_R\delta n_e/n_e$, where

$f = \bar{E}_L = \alpha_R \delta n_e/n_e$ is needed for equations (3)-(5). The solution to this apparent paradox is provided in Refs. [38, 39]: here, the electrostatic field is explicitly given in the form $\bar{E}_L = f\exp(i\theta) + \text{c.c.} = 2f\cos(\theta)$, and similarly $\delta n_e/n_e = 2\tilde{f}\cos(\theta)$, or $\tilde{f} = \alpha_R \delta n_e/n_e$ after scaling. So when it is stated that "f denotes the scaled electrostatic potential/field/density fluctuation", it may well mean that f denotes *half* that quantity. Further support for this is found in Refs. [40, 41], where the Manley-Rowe relation $||a_0||^2\omega_0 = 2g(||\bar{E}_L||/2)^2\omega_{pe}$ has been derived using an energy-conservation argument rather than a set of three-wave equations. Comparing this to the Manley-Rowe relation $||a_0||^2\omega_0 = 2g||f||^2\omega_{pe}$ by Malkin *et al.* [33], we find that this model must satisfy $f = \bar{E}_L/2$ in order to conserve energy. Compatibility with the model by Forslund *et al.* then follows by default, and the posted wave breaking limit for circular polarisation, $a_{wb} = (\omega_{pe}/\omega_0)^{3/2}/4$, is obtained for $\delta n_e/n_e = 1$, as it should. While this does not affect the current discussion, it will become important when systems of three-wave equations are used that contain higher-order terms in f.

Following Malkin, Shvets and Fisch [33], or Menyuk, Levi and Winternitz [42], we define $\zeta = \Gamma_R a_{00}(x/c+t)$, $t' = \Gamma_R a_{00} t$ and $\xi = 2\sqrt{\zeta t'}$ where a_{00} denotes the pump pulse amplitude. Attractor solutions to the system (3)-(5) can then be obtained in terms of ξ alone. In particular, the first peak of the growing seed pulse can be approximated by $a_1(\zeta,t') \approx (2a_{00}t'/\xi)\partial u(\xi)/\partial\xi$ where $u(\xi) = 2\sqrt{2}\arctan[\epsilon\exp(\xi)/(4\sqrt{2\pi\xi})]$, with $\epsilon < 0.1$ depending on the initial seed pulse B-integral $\int a_1(\zeta,0)d\zeta$. The function $\partial u(\xi)/\partial\xi$ has an amplitude $A \approx 1.29$ and a width $\Delta\xi \approx 2.65$, mostly independent of ϵ, while the position of its maximum, ξ_M, obeys $5 < \xi_M < 7$ for practical values of ϵ [33, 43]. Let $\Delta\zeta$ denote the width of the first peak of $a_1(\zeta,t')$ for fixed t' and let $\xi_{1,2} = \xi_M \pm \Delta\xi/2$. Then $\xi_M\Delta\xi = (\xi_2^2 - \xi_1^2)/2 = 2t'\Delta\zeta$. For $\xi = \xi_M$, we find that $||a_1|| = 2Aa_{00}t'/\xi_M$, $\Delta\zeta = \xi_M\Delta\xi/(2t')$. We consider pump and seed pulses with durations τ_0 and τ_1 (after amplification), and setting $\Delta\zeta = \Gamma_R a_{00}\tau_1$ and $t = \tau_0/2$ (for an interaction time t, the seed pulse sees a length $\sim ct$ of plasma column, but since the pump pulse is advancing at speed $-c$, the seed effectively interacts with $2t$ of pump pulse), we find:

$$\Gamma_R^2 a_{00}^2 \tau_0\tau_1 = \xi_M\Delta\xi \approx 15, \tag{6}$$

$$\Gamma_R||a_1||\tau_1 = A\Delta\xi \approx 3.4. \tag{7}$$

The scalings for the seed pulse amplitude and duration are $||a_1||(t) = (2A/\xi_M)a_{00}^2\Gamma_R t$, $\Gamma_R\tau_1(t) = \xi_M\Delta\xi/(2a_{00}^2\Gamma_R t)$. The asymptotic energy transfer efficiency for the first peak is given by $\eta = ||a_1||^2\tau_1(t)/(2a_{00}^2 t) = A^2\Delta\xi/\xi_M \approx 4.4/\xi_M$. Thus, η is constant for a given configuration, and decreases with increasing ξ_M.

The strength of these new relations is also shown in their potential to obtain a deeper understanding of earlier work. For example, in a paper by Malkin and Fisch on Raman amplification in the presence of plasma wave damping [44], it is suggested that the damping can be overcome by increasing the initial seed pulse amplitude. However, no explicit expression for the minimum seed pulse amplitude necessary is given in Ref. [44]. With the aid of Eqns. (6) and (7), we can now derive that threshold. From Eqns. (18)-(20) of Ref. [44], we obtain (using our own definitions of ζ and Γ_R): $a_{00}\Gamma_R\partial_\zeta b + \nu b = \Gamma_R a_0 a_1$. Clearly, for nonlinear amplification to dominate over damping, we must have $|a_{00}\Gamma_R\partial_\zeta b| > |\nu b|$. From Eq. (7) above, we obtain $||a_{00}\Gamma_R\partial_\zeta|| \propto 1/\tau_1 \propto \Gamma_R||a_1||$; we take $|a_{00}\Gamma_R\partial_\zeta b| \sim \Gamma_R||a_1||b$ as a first approximation. Immediately we find $\Gamma_R||a_1|| > \nu$ as the criterion for non-linear Raman amplification to dominate over Langmuir wave damping. For a constant damping coefficient, e.g. $\nu = q\Gamma_R a_{00}$, amplification will overcome damping if $||a_1||/a_{00} > q$. This is directly borne out by figures 2-4 of Ref. [44], where non-linear amplification truly takes off as soon as a_1^2/a_{00}^2 exceeds q^2 (25, 100 and 400 respectively). For a linearly growing damping coefficient, e.g. $\nu = q_\nu\Gamma_R^2 a_{00}^2 t$, we apply the seed pulse scaling $||a_1||(t) = (2A/\xi_M)a_{00}^2\Gamma_R t$. We find that damping dominates over amplification if $\Gamma_R||a_1||(t) = (2A/\xi_M)a_{00}^2\Gamma_R^2 t < q_\nu\Gamma_R^2 a_{00}^2 t$, or $q_\nu > 0.45$. This is directly borne out by Figure 1 of Ref. [44], where true nonlinear amplification (including secondary peaks in the seed) can only be seen for those cases where $q_\nu < 0.5$. The ease with which we can derive these thresholds and extend the theory of Ref. [44] is a clear demonstration of the power of our equations (6) and (7).

B. Brillouin amplification

Brillouin amplification in plasma is based on principles similar to Raman scattering, only the Langmuir plasma wave is replaced by an ion-acoustic wave. Because the frequency of the ion-acoustic wave will be much smaller than the frequency of the pump laser, pump and seed laser beams can have (nearly) the same frequency. Brillouin scattering in the so-called weak-coupling regime [31, 32, 34, 45–49] is very similar to Raman scattering and can be treated in the same way. We introduce $\omega_{pi} = \omega_{pe}\sqrt{Zm_e/m_i}$ and $c_s = v_e\sqrt{Zm_e/m_i}$. For $a_{00}^2 < 8g(\omega_0/\omega_{pe})^2\sqrt{Zm_e/m_i}v_e^3/c^3$, the electron pressure is the dominant restoring force and the plasma wave dispersion is not significantly affected by the beating between pump and seed pulses. In that case one can reuse equations (3)-(5) and only needs to replace $3v_e^2(k/\omega_{pe})$ by c_s in Eq. (5). For backward Brillouin scattering, the ion-acoustic wave has wave number $k_s \approx 2k_0$ and frequency $\omega_s = c_s k_s \approx 2c_s k_0$. Then we find $\Gamma_B\alpha_B = \omega_{pe}^2/(4\omega_0)$ and $\Gamma_B/\alpha_B = c^2c_s^2k_s^2/(4g\omega_s v_e^2)$, leading to

$\Gamma_B = c\omega_{pe}\omega_s/(4v_e\sqrt{g\omega_0\omega_s})$ and $\alpha_B = \sqrt{g}v_e\omega_{pe}/(c\sqrt{\omega_0\omega_s})$. After substituting Γ_B, α_B for Γ_R, α_R, all the above results for Raman amplification also apply to the weak-coupling Brillouin case, including Eqns. (6) and (7), the wave breaking threshold $a_{wb} = \alpha_B/\sqrt{2}$, the numerical constants $5 < \xi_M < 7$, $\Delta\xi \approx 2.65$ and $A \approx 1.29$, and the seed pulse scalings.

For $a_{00}^2 > 8g(\omega_0/\omega_{pe})^2\sqrt{Zm_e/m_i}v_e^3/c^3$, or $\Gamma_B a_{00}/\omega_0 > c_s/c$, the ponderomotive pressure from the beating between pump and seed pulses will take over from the thermal pressure as the primary restoring force for the ion-acoustic wave. In this regime, called *strong-coupling* (sc) Brillouin scattering, the equation for the plasma wave becomes [31, 50, 51]:

$$\partial^2 b/\partial t^2 = -\alpha_{sc}c^2k^2\omega_{pi}^2/(2g\omega_{pe}^2)a_0a_1 = -\Gamma_{sc}^2 a_0 a_1. \tag{8}$$

Here, Γ_{sc} denotes the coupling constant for sc-Brillouin backward scattering. From (3) and (8) and using $k = 2k_0 \approx 2\omega_0 v_g/c^2$ as before, we find: $\Gamma_{sc}\alpha_{sc} = \omega_{pe}^2/(4\omega_0)$ and $\Gamma_{sc}^2/\alpha_{sc} = c^2k^2\omega_{pi}^2/(2g\omega_{pe}^2)$. This yields [31, 50, 51]:

$$\Gamma_{sc} = [(v_g/c)^2\omega_{pi}^2\omega_0/(2g)]^{1/3} = (2\omega_s\Gamma_B^2)^{1/3}, \tag{9}$$

$$\alpha_{sc} = \omega_{pe}^2/(4\omega_0\Gamma_{sc}), \tag{10}$$

$$\Omega_{sc} = \omega_{sc} + i\gamma_{sc} = [(1+i\sqrt{3})/2]\Gamma_{sc}a_{00}^{2/3}, \tag{11}$$

where ω_{sc} and γ_{sc} denote the frequency and growth rate of the driven ion wave. The numerical factor for Ω_{sc} must be a (complex) cube root of -1, and must have a positive imaginary part for the plasma wave to grow rather than damp, and the only suitable value is then $(1+i\sqrt{3})/2$.

To make the connection with earlier work: if one uses $\langle a_{0,1}\cdot\delta n_e\rangle = a_{0,1}\delta n_e/2$ and $\nabla^2\langle A^2\rangle = -(2k_0)^2 a_0 a_1/g$ in Hüller *et al.* [50], then this work agrees with Forslund *et al.* [31]. In Refs. [52, 53], all quantities are $\propto \exp(i\theta)$ rather than $\propto \cos(\theta)$, so both $\langle a_{0,1}\cdot\delta n_e\rangle$ and $\langle a_0\cdot a_1\rangle$ are twice what they should have been. Furthermore, Refs. [52, 53] use $\nabla^2\langle a_0\cdot a_1\rangle = -k_0^2\langle a_0\cdot a_1\rangle$ rather than $\nabla^2\langle a_0\cdot a_1\rangle = -(2k_0)^2\langle a_0\cdot a_1\rangle$ for unknown reasons. As a result, Refs. [52, 53] cite the correct value for Γ_{sc} (for linear polarisation), but their value for α_{sc} is twice what it should have been. This appears to have been corrected by Schluck *et al.* [51] though, whose equations agree with Forslund *et al.* [31].

Following the approach by Andreev *et al.* [52], we define $t' = \Gamma_{sc}a_{00}^{2/3}t$, $\zeta = \Gamma_{sc}a_{00}^{2/3}(t + x/v_g)$ and $\xi = \zeta\sqrt{t'}$. Again, attractor solutions to the system (3), (4) and (8) can be obtained in terms of ξ alone. In particular, the resulting seed pulse will scale as $a_1(\zeta, t') = a_{00}(t')^{3/4}f(\xi)$ where $f(\xi)$ has a fixed duration $\Delta\xi \approx 3.3$ and an amplitude $A \approx 0.62$ [52, 53]. Using $\Delta\xi = (\Delta\zeta)\sqrt{t'}$ for fixed t', $||a_1|| = a_{00}A(\Delta\xi/\Delta\zeta)^{3/2}$ and inserting $\Delta\zeta = \Gamma_{sc}a_{00}^{2/3}\tau_1$ and $t' = \Gamma_{sc}a_{00}^{2/3}\tau_0/2$ for pump and seed pulses with durations τ_0 and τ_1 into $\Delta\xi$ yields:

$$\Gamma_{sc}^3 a_{00}^2\tau_0\tau_1^2 = 2(\Delta\xi)^2 \approx 22, \tag{12}$$

$$\Gamma_{sc}^3||a_1||^2\tau_1^3 = A^2(\Delta\xi)^3 \approx 13.8. \tag{13}$$

The scalings for the seed pulse amplitude and duration are then $||a_1||(t) = A(a_{00}^2\Gamma_{sc}t)^{3/4}$ and $\Gamma_{sc}\tau_1(t) = \Delta\xi/(a_{00}^2\Gamma_{sc}t)^{1/2}$, and the asymptotic efficiency is $\eta = a_1^2(t)\tau_1(t)/(2a_{00}^2 t) = A^2\Delta\xi/2 \approx 0.63$.

C. Properties of the equations equations (7) and (13)

As discussed in the main manuscript, the equations (7) and (13) are fully valid only when the seed pulse amplitude exceeds certain thresholds, ruling out the case of a very long initial seed pulse with a very low amplitude. The first threshold follows from the fact that the various models for Raman and Brillouin amplification require fully nonlinear interaction and full pump depletion. The "self-similar" model for Raman amplification by Malkin, Shvets and Fisch [33] has been derived under the condition that $\partial a_1/\partial\zeta > \partial a_1/\partial t$. We use the scalings $\partial a_1/\partial t \propto \Gamma a_0^2$ and $\partial a_1/\partial\zeta \propto \Gamma a_1^2$ to arrive at the threshold condition for the seed amplitude $a_{10} > a_{00}$. For sc-Brillouin amplification [52], we use the scalings $\partial a_1/\partial t \propto a_{00}^2\Gamma/(a_{00}^2\Gamma t)^{1/4} \propto a_{00}^2\Gamma/a_1^{1/3}$ and $\partial a_1/\partial\zeta \propto \Gamma a_1^{5/3}$ to again arrive at $a_{10} > a_{00}$. All this means that our criterion works best if the initial seed pulse intensity is at least that of the pump beam.

Phenomena like plasma wave damping or chirping of the frequency of either the pump beam or the plasma wave can impose further thresholds. To derive the seed amplitude threshold in case of damping, we start from an initial seed pulse with amplitude a_1 and duration τ_1 satisfying Eq. (7). From Eq. (18) and (20) of Ref. [44], we find the equation for the damped plasma wave $\partial_\zeta b - \nu b. = \Gamma_R a_0 a_1$. We insert the initial seed pulse into this equation and require that growth dominates over damping, i.e. $|\Gamma_R a_0 a_1| > |\nu b|$. Requiring full pump depletion, i.e. $||a_0|| \sim ||b||$, we then obtain the condition $\Gamma_R|a_1| > \nu$ for a viable seed pulse. This means that the "damping distance" $1/\nu$ is larger than the "pump depletion distance" $1/(\Gamma_R a_1)$. For the case of chirp, we use Eq. (1) of Ref. [54] to obtain the equation for the plasma wave $\partial_\zeta b - i(\delta\omega)b = \Gamma_R a_0 a_1$. At the start of the interaction, we have $\delta\omega = q\tau_1 \sim q/(\Gamma_R a_1)$.

We require that growth dominates over detuning, i.e. $|\Gamma_R a_0 a_1| > q|b|/(\Gamma_R a_1)$. Again requiring $||a_0|| \sim ||b||$, we obtain the condition $\Gamma_R^2 a_1^2 > q$ for a viable seed pulse. This means that the detuning $\delta\omega = q\tau_1 \sim q/(\Gamma_R a_1)$ due to chirp over the duration of the seed pulse is smaller than the bandwidth $1/\tau_1 \sim \Gamma_R a_1$ of the seed pulse. The seed amplitude thresholds for sc-Brillouin amplification are obtained using similar arguments. We note that we could not have derived these thresholds without using Eq. (7), which underlines its importance.

The salient properties of equations (7) and (13), and the equivalent relations (1) and (2) for $\Gamma_R^2 \mathcal{E}_1 \tau_1$ and $\Gamma_{sc}^3 \mathcal{E}_1 \tau_1^2$, are as follows. (i) These relations have been derived for asymptotic attractor solutions for the seed pulse, and $\Gamma_R \tau_1 a_1$ or $\Gamma_{sc} \tau_1 a_1^{2/3}$ will therefore only settle on their asymptotic values after a significant interaction distance. The specific distance needed varies significantly with the initial seed pulse size and shape, and can be an object of further study. (ii) The triple products $\Gamma_R \tau_1 a_1$ and $\Gamma_{sc} \tau_1 a_1^{2/3}$ are *not constants of motion* and will vary in particular during the early stages of the interaction. Their values can be used to assess how close the seed pulse is to the asymptotic π-pulse solution. For this reason, curve fitting experimental data pairs $(\mathcal{E}_1, \tau_1)$ with a curve of the form $\tau_1 = \alpha \mathcal{E}_1^{-\beta}$ does not work, since it assumes that all output seed pulses are perfect π-pulses already, while the seed pulses usually do not fully reach that stage within the limited interaction length available. Instead, one should simply use the value of the triple product $\Gamma_R \tau_1 a_1$ to judge how well a given output pulse resembles a perfect π-pulse. (iii) It is often stated that the π-pulse solution is an attractor [33], even if no complete mathematical proof is ever given. However, the asymptotic values for $\Gamma_R \tau_1 a_1$ and $\Gamma_{sc} \tau_1 a_1^{2/3}$ definitely have attractor properties, as borne out by the simulation results in the main manuscript. (iv) Since the equations (7) and (13) are completely independent of the pump pulse, they will continue to hold under circumstances not covered by the original "self-similar" models, e.g. slowly varying pump amplitude or plasma density, two or three dimensions (see below), higher order laser modes such as Laguerre-Gaussian (see below), full numerical simulations or experiments that know nothing about the approximations needed to arrive at the π-pulse solutions of Refs. [33, 52]. (v) Following Ref. [33], we define $\epsilon = \Gamma_R \int d\zeta g(t = 0, \zeta)$, the integrated amplitude of the initial seed pulse. For weak seed pulses, $\epsilon \ll 1$. We then find that the position of the first seed pulse maximum is given by $\xi_M \approx 2.31 + \ln(1/\epsilon)$. Even though the dependence of ξ_M on ϵ is "only logarithmic", we find that the intensity of seed pulses used in experiments, and thus ϵ, can vary by orders of magnitude, leading to significant shifts in ξ_M, as well as in the first-peak efficiency $4.4/\xi_M$. Since for a finite interaction length the first peak may well be the only one that develops fully (the long tail of trailing peaks needs "infinite" time to develop, see below), this explains why a poor choice for the initial seed pulse can have a lasting negative impact on the efficiency, as shown in Figure 3 of the main manuscript.

It is important to remember that Malkin *et al.* and Andreev *et al.* provide asymptotic solutions for the growing seed pulse that are valid in the limit $t \to \infty$, while experiments and most numerical simulations are carried out during a limited, finite interaction time. When a realistic seed pulse evolves towards the asymptotic solution, the head of the pulse will form first, and the tail of secondary pulses only after that. This can be quantified as follows. From the definitions of the "self-similar" coordinates ξ above, we find for Raman amplification: $\partial_\zeta = \sqrt{t'/\zeta}\partial_\xi$, $\partial_{t'} = \sqrt{\zeta/t'}\partial_\xi = (\zeta/t')\partial_\zeta$. Since the π-pulse solution has been derived under the assumption $\partial_{t'} \ll \partial_\zeta$, e.g. $\partial_{t'} \lesssim 0.1\partial_\zeta$ this means that for a given distance ζ behind the head, the pulse will only begin to resemble the π-pulse for e.g. $t' \gtrsim 10\zeta$. Thus, in realistic situations the leading spike of the growing seed pulse will form first, and the "tail" of subsequent spikes will form much later, if at all. (See e.g. Figure 3 of Ref. [6], where the head of the seed pulse is fully formed but the tail much less so.) This means that when analysing seed pulses from experiments, one cannot offhand assume that a tail of trailing spikes exists and contains a fixed fraction of the total pulse energy. After the limited interaction distance available to many experiments, the amplified seed pulse may consist of the leading spike only. Similarly, we find for strongly coupled Brillouin amplification that $\partial_\zeta = \sqrt{t'}\partial_\xi$, $\partial_{t'} = (\zeta/2\sqrt{t'})\partial_\xi = (\zeta/2t')\partial_\zeta$, so $\partial_{t'} \lesssim 0.1\partial_\zeta$ implies $t' \gtrsim 5\zeta$ for a given distance ζ behind the head of the seed pulse. In general, if one requires $|\partial_{t'}| < \varepsilon|\partial_\zeta|$ for some $\varepsilon \ll 1$, then this only holds for $0 < \zeta < \varepsilon t'$ or $0 < \xi < 2t'\sqrt{\varepsilon}$ for Raman amplification, and for $0 < \zeta < 2\varepsilon t'$ or $0 < \xi < 2\varepsilon(t')^{3/2}$ for sc-Brillouin amplification.

The difference between "transient" and "asymptotic" solutions for the seed pulse is also important in the discussion surrounding the duration, amplitude and shape of the "optimal" seed pulse, especially when a seed pulse with a duration shorter than "optimal" still leads to the highest amplification efficiency (see figure 3 in the main manuscript). We note that a truly "optimal" seed pulse does not only have the optimal relationship between amplitude and duration, but also the ideal π-pulse shape with a tail of secondary peaks. This is impossible to generate in an experiment, so we did not use the ideal π-pulse shape in our simulations either; we used simple Gaussian pulse envelopes instead. When using Gaussian pulse shapes with varying values for $\Gamma_R \tau_1 a_1$, it may be that the pulse that reshapes to the optimal π-pulse fastest has an initial value for $\Gamma_R \tau_1 a_1$ that differs somewhat from 3.4, even if the long-term value for $\Gamma_R \tau_1 a_1$ will always settle on 3.4. Even so, the "optimal" value for $\Gamma_R \tau_1 a_1$ in our simulations is still close enough to 3.4 to make our criteria very valuable for designing experiments, especially since the values for $\Gamma_R \tau_1 a_1$ of previous experiments are always so far below 3.4 (see discussion in the Supplemental Material).

In summary, the main gap between the models by Malkin *et al.* [33] and Andreev *et al.* [52] on one hand, and

real-life experimental situations on the other, is that the experimental results are mainly determined by "transient" solutions to the three-wave equations while such "transient" solutions are not studied in the above two models at all. Our work aims to (i) bridge this gap by looking at transient behaviour of the seed pulse, and (ii) determine the necessary conditions to get the initial seed pulse as close to the asymptotic solution as possible, in order to make the most of the limited interaction distance and boost the overal efficiency of the amplification. Furthermore, the above-mentioned models are all one-dimensional. While direct Raman or Brillouin backscattering are technically 1-D phenomena, an 1-D model is not enough to capture and explain all the dynamics of Raman/Brillouin amplification using finite-width pulses. As argued above, filamentation and self-focusing play a vital role in the success or failure of any Raman/Brillouin amplification experiment. As shown above, configurations with weak seed pulses suffer more from filamentation and self-focusing than configurations with strong seed pulses, underlining how important the power of the initial seed pulse is to the success of an experiment. None of this is covered by existing 1-D models.

III. THERMAL EFFECTS

Plasma distinguishes itself from other non-linear media by the importance of thermal effects. While thermal effects can often be neglected in Raman amplification in e.g. non-linear fibers, thermal effects must be considered in plasma because all plasma is necessarily thermal, or it would recombine to neutral gas.

Thermal effects are automatically included in the study of Brillouin amplification, since the ion-acoustic wave involved requires thermal pressure to even exist. The plasma temperature controls both the frequency of the ion-acoustic wave and the growth rate of the process in weakly coupled Brillouin amplification. This is already discussed in the main manuscript. In strongly coupled Brillouin amplification, the beating between the EM waves dominates over the thermal plasma pressure, so thermal effects only play a minor role in this process. Thermal effects need to be studied in Raman amplification in particular, since Raman amplification can happen in both cold and warm plasma, but it is more complicated in warm plasma and the behaviour of Raman scattering in cold and warm plasma is not completely the same.

Thermal effects can influence Raman amplification in plasma in three main ways: (i) reduction of the wave breaking limit of the Langmuir wave, (ii) increase of the Langmuir wave frequency via the Bohm-Gross dispersion relation, and (iii) Landau damping of the Langmuir wave.

Wave-breaking limit For a non-relativistic Langmuir wave in cold plasma, the wave breaking limit is given by $eE_L/(m_e\omega_{pe}v_\varphi) = 1$, where E_L is the electric field amplitude of the Langmuir wave, v_φ its phase speed, and other symbols have their usual meaning [35]. For a non-relativistic Langmuir wave in warm plasma, the wave breaking limit is given by $eE_L/(m_e\omega_{pe}v_\varphi) = (1 - \frac{8}{3}\beta^{1/4} + 2\beta^{1/2} - \frac{1}{3}\beta)^{1/2}$, where $\beta = 3k_BT_e/(m_e v_\varphi^2)$ and T_e is the plasma electron temperature [56]. In Raman backscattering, the Langmuir wave has a phase speed $v_\varphi/c = \omega_{pe}/(2\omega_0)$, so $\beta = 12(k_BT_e/m_ec^2)(\omega_0^2/\omega_{pe}^2)$. This likewise reduces the pump pulse amplitude limit for wave breaking: $a_{wb} = (\alpha_R/\sqrt{2})(1 - \frac{8}{3}\beta^{1/4} + 2\beta^{1/2} - \frac{1}{3}\beta)^{1/2}$. For a pump amplitude above this limit, the Langmuir wave induced by Raman scattering will break and the scattering process will evolve into Stimulated Compton Scattering [57], which obeys entirely different scaling laws. Thus, one has to take care that one's pump amplitude is below the warm-plasma limit when considering Raman amplification in warm plasma, not just below the cold-plasma limit.

Bohm-Gross dispersion The dispersion relation for a Langmuir wave in cold plasma reads: $\omega_L^2 = \omega_{pe}^2$ [35]. The dispersion relation for a Langmuir wave in warm plasma reads: $\omega_L^2 = \omega_{pe}^2 + 3(k_BT_e/m_e)k_L^2$ [58]. For Raman backscattering in plasma, one has $k_L \approx 2\omega_0/c$ and thus $\omega_L^2 = \omega_{pe}^2 + 12(k_BT_e/m_e)\omega_0^2/c^2 = \omega_{pe}^2[1 + 12(k_BT_e/m_e)(\omega_0^2/\omega_{pe}^2)] = \omega_{pe}^2(1+\beta)$. For e.g. $k_BT_e = 100$ eV and $\omega_0/\omega_{pe} = 15$, one finds that $\omega_L = 1.24\omega_{pe}$. Since the frequency ω_1 of the signal pulse is determined via $\omega_1 = \omega_0 - \omega_L$, this leads to a temperature-induced shift in ω_1 even if ω_0 and ω_{pe} are unchanged. One has to take this shift into account when setting up the initial configuration for Raman amplification.

Landau damping Landau damping [59, 60] is the collisionless damping of longitudinal plasma waves, involving the conversion of plasma wave energy into plasma thermal energy. Raman amplification will be affected by Landau damping via the damping of the Langmuir wave, although this damping will saturate if the Raman process is sufficiently intense [61–63]. For Raman amplification, Landau damping of the electron plasma wave becomes significant when its phase speed becomes comparable to the electron thermal speed, i.e. when $\omega_{pe}/(2\omega_0) \lesssim 3v_{Te}/c$, or $T_e \gtrsim 0.025(n_0/n_{cr})$[511keV]. For $(n_0/n_{cr}) = 1/400$, we find significant Landau damping for $T_e \gtrsim 100$ eV. Such temperatures have been reported in past experiments with long pump beams [7, 8]. For sc-Brillouin amplification, assuming that $T_i \sim T_e$, we find that the phase speed of the driven ion mode is larger than the ion thermal speed but smaller than the electron thermal speed, so neither ions nor electrons can cause significant Landau damping of the plasma wave.

To describe the effect of saturated Landau damping we use the model by Yampolsky and Fisch [64]. The procedure

works as follows. First, we determine the Landau damping rate using the following textbook expression:

$$\gamma_L = -\left(\frac{\pi}{8}\right)^{1/2}\frac{\omega_{pe}}{(k_L\lambda_D)^3}\exp\left[-\frac{1}{2(k_L\lambda_D)^2}-\frac{3}{2}\right],$$

where $k_L\lambda_D = 2(\omega_0/\omega_{pe})(v_T/c)$ and $v_T^2 = k_BT_e/m_e$.

Second, we solve Eq. (29) from Ref. [64] to calculate the effective pump amplitude $\tilde{a}_{00}$ from the original pump amplitude a_{00}:

$$\tilde{a}_{00}^2 + (\omega_{pe}/\omega_0)^{3/4}(\gamma_L/\omega_{pe})\tilde{a}_{00}^{3/2} = a_{00}^2.$$

Third, we observe that the model used in Eqns. (30)-(34) of Ref. [64] is very similar to the cold-plasma model used by Malkin, Shvets and Fisch [33], except that $t' = \Gamma_R\tilde{a}_{00}^2 t$ is used instead of $t' = \Gamma_R a_{00}^2 t$. Eq. (4) of the main manuscript then becomes:

$$\Gamma_R||a_1||\tau_1 \approx 3.4(a_{00}/\tilde{a}_{00}). \tag{14}$$

Note that the influence of Landau damping on Eq. (3) of the main manuscript is harder to determine, since Landau damping increases the value of the constant ξ_M too, and no analytical expression for this increase is supplied.

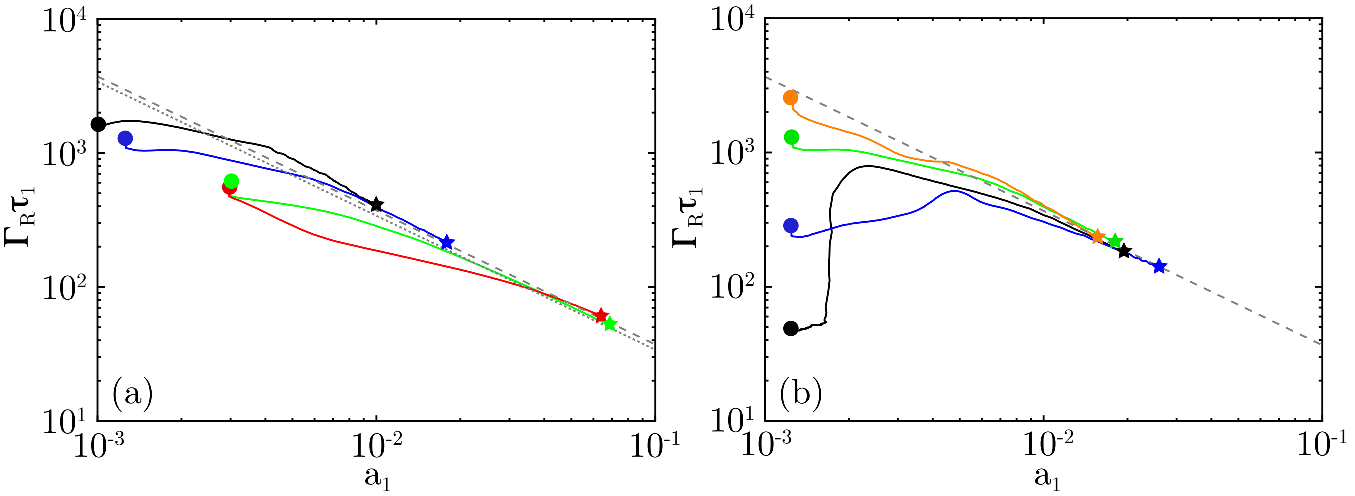

Figure 3: Analysis of particle-in-cell simulations of Raman amplification in a warm plasma. Shown are $\Gamma_R\tau_1$ versus a_1 for various values of k_BT_e, n_0/n_{cr} and a_0. Frame (a) shows the evolution of the seed pulse in Raman amplification with fixed initial pulse duration and varying pulse intensities, while frame (b) shows the evolution of the seed pulse for fixed intensity but varying seed pulse duration. Simulation parameters are as given in the text. The dashed lines correspond to Eqns. (14). The evolution of each curve starts at the circle and ends at the star. These results show clearly that for a warm plasma, just like for a cold plasma, the triple product $\Gamma_r||a_1||\tau_1$ tends toward a constant for the growing signal pulse, irrespective of pulse intensity or initial pulse duration. While thermal corrections provide a minor correction (up to 10%) to the numerical value of this constant, there is no qualitative change to the concept at all.

We calculate the factor $a_{00}/\tilde{a}_{00}$ for various combinations of a_{00}, ω_0/ω_{pe} and k_BT_e:

a_{00}	ω_0/ω_{pe}	k_BT_e (eV)	$a_{00}/\tilde{a}_{00}$
0.010	10	100	1.095
0.010	10	200	1.790
0.015	10	100	1.078
0.015	10	200	1.652
0.032	15	12	1.000
0.032	15	50	1.101
0.032	15	100	1.592
0.032	15	200	2.602

We find that the effect of Landau damping decreases with a_{00}, as long as there is no Langmuir wave breaking, since Landau damping saturates sooner when the Raman backscattering process is driven harder. The effect increases with increasing ω_0/ω_{pe}, because this lowers the phase speed of the Langmuir wave and makes it more susceptible to damping.

To verify the above model, we have conducted a number of particle-in-cell simulations of Raman amplification in warm plasma. The results are displayed in Figure 3. Parameters are as follows: Figure 3(a): $k_BT_e = 100$ eV, $n_0/n_{cr} = 0.01$ and $a_0 = 0.01$ (black) or 0.015 (blue); $a_0 = 0.032$, $n_0/n_{cr} = 0.0044$ and $k_BT_e = 12$ eV (green) or 50 eV (red). Figure 3(b): $k_BT_e = 100$ eV, $n_0/n_{cr} = 0.01$ and $a_0 = 0.015$, with τ_1/τ_R the same as in Figure 2(b) in the main manuscript. Parameters not listed are as given in the main manuscript. The dashed lines correspond to Eqns. (14) for Raman, (which is like Eq. (4) in the main manuscript with a 1.1 thermal correction factor).

Once again, we find that the evolving seed pulses closely follow the analytical predictions, irrespective of the pump intensity chosen in the simulations, proving that the non-linear matching conditions for Raman amplification with thermal correction, given by (14), still represents an "attractor" solution and remains valid over a wide range of pulse intensities, and that thermal effects do not make any qualitative change to our findings.

IV. TRANSVERSE EFFECTS

In a multi-dimensional setting, the amplitude a_1 and duration τ_1 of the growing seed laser pulse will of course depend on the transverse coordinate x_2. The same holds true for the location of the seed pulse maximum ζ_M in a frame comoving with the pulse. This leaves an imprint on the full shape of the envelope when parametric amplification is studied in more than one dimension.

We recall that the seed pulse amplitude and duration equation (7) for Raman amplification, and equation (13) for strongly coupled Brillouin amplification respectively (see above). We find that the x_2-dependence of the amplitude a_1 also induces an x_2-dependence of the duration τ_1, even for fixed t. For example, for seed pulses with a Gaussian envelope, $a_1(x_2) \propto \exp(-x_2^2/w_0^2)$, this results in a horseshoe envelope $\tau_1(x_2) \propto \exp(x_2^2/w_0^2)$ [36, 43, 65–71]. For donut-shaped seed pulses with orbital angular momentum, this even results in a "double horseshoe" envelope [72]. We will verify such horseshoe seed pulse shapes against Eqns. (7) and (13) here.

In Figure 4, we verify the horseshoe shape of seed pulses after amplification. Here, we present the analysis of a 2-D Raman simulation (Figures 4(a) and 4(d)), taken from Ref. [70], Figure 2a, a 3-D Raman simulation with orbital angular momentum and a doughnut-shaped seed pulse intensity envelope (Figures 4(b) and 4(e)), taken from Ref. [72], Figure 1a and 2a, and a 2-D sc-Brillouin simulation (Figures 4(c) and 4(f)), using $m_i/(Zm_e) = 1836$, $n_0/n_{cr} = 0.3$ and $a_0 = 0.027$. Figures 4(a)-(c) show the vector potential envelopes for these pulses. Figures 4(d)-(f) show $a_1(x_2)$, $\omega_0\tau_1(x_2)$ and $\Gamma_R a_1(x_2)\tau_1(x_2)$ and $A\Delta\xi \approx 3.4$ (Figure 4(d) and 4(e)) or $\Gamma_{sc}^3 a_1^2(x_2)\tau_1^3(x_2)$ and $A^2\Delta\xi^3 \approx 13.8$ (Figure 4(f)), all versus transverse coordinate x_2. In all cases, the curved shape of the seed pulse is well described by Eqns. (7) or (13). Even for strange pulse envelope shapes, like the "donut" shape of the pulse with OAM, the relationships between pulse amplitude and duration still hold. The presence or absence of parasitic instabilities does not have much of an influence on this behaviour. For example, in Ref. [70], one can see numerical simulations of two Raman-amplified laser pulses, one with and one without filamentation. Both pulses exhibit the horseshoe shape predicted here.

[1] Y. Ping *et al.*, Phys. Rev. E **66**, 046401 (2002).
[2] Y. Ping *et al.*, Phys. Rev. Lett. **92**, 175007 (2004).
[3] W. Cheng *et al.*, Phys. Rev. Lett. **94**, 045003 (2005).
[4] D. Turnbull *et al.*, Phys. Plasmas **19**, 073103 (2012).
[5] D. Turnbull *et al.*, Phys. Plasmas **19**, 083109 (2012).
[6] J. Ren *et al.*, Nature Phys. **3**, 732 (2007).
[7] R. Kirkwood *et al.*, Phys. Plasmas **14**, 113109 (2007).
[8] Y. Ping *et al.*, Phys. Plasmas **16**, 123113 (2009).
[9] R. Kirkwood *et al.*, J. Plasma Phys. **77**, 521 (2011).
[10] G. Vieux *et al.*, Proc. SPIE **7359**, 73590R (2009).
[11] G. Vieux *et al.*, New J. Phys. **13**, 063042 (2011).
[12] X. Yang *et al.*, Sci. Rep. **5**, 13333 (2015).
[13] A. Balakin *et al.*, JETP Lett. **80**, 12 (2004).
[14] M. Dreher *et al.*, Phys. Rev. Lett. **93**, 095001 (2004).
[15] C.-H. Pai *et al.*, Phys. Rev. Lett. **101**, 065005 (2008).
[16] P. F. Moulton, J. Opt. Soc. Am. B **3**, 125 (1986).
[17] J. Ren *et al.*, Phys. Plasmas **15**, 056702 (2008).

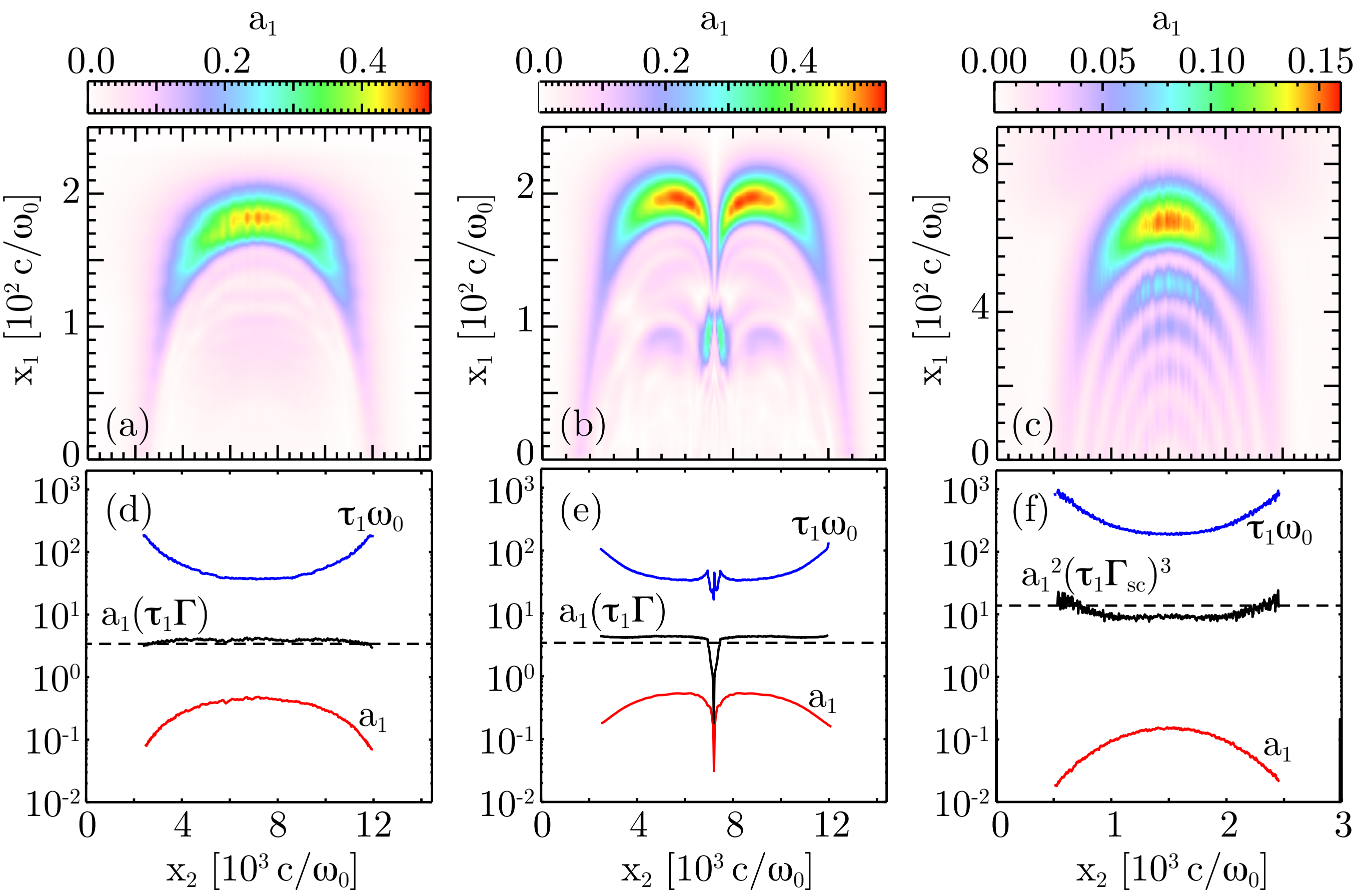

Figure 4: Analyzing the curved shape of the amplified seed pulse for Raman (a,d), Raman with OAM (b,e) (taken from Refs. [70, 72]) and Brillouin (c,f) amplification in multi-dimensional simulations, taken from Refs. [70, 72]. Frames (a)-(c) show the amplitude envelopes of the amplified seeds. Frames (d)-(f) show $a_1(x_2)$ (red) and $\omega_0\tau_1(x_2)$ (blue) versus the transverse coordinate x_2. The products $a_1(x_2)\Gamma_R\tau_1(x_2)$ in frames (d) and (e), and $a_1^2(x_2)[\Gamma_{sc}\tau_1(x_2)]^3$ in frame (f) (solid black curves) are then verified against Eqns. (7) and (13) (dashed lines).

[18] N. A. Yampolsky *et al.*, Phys. Plasmas **15**, 113104 (2008).
[19] R. H. Lehmberg and S. P. Obenschain, Opt. Comm. **46**, 27 (1983).
[20] S. P. Obenschain *et al.*, Phys. Rev. Lett. **56**, 2807 (1986).
[21] J. Grun *et al.*, Phys. Rev. Lett. **58**, 2672 (1987).
[22] A. N. Mostovych *et al.*, Phys. Rev. Lett. **59**, 1193 (1987).
[23] S. P. Obenschain *et al.*, Phys. Rev. Lett. **62**, 768 (1989).
[24] T. A. Peyser *et al.*, Phys. Plasmas **3**, 1479 (1991).
[25] J. E. Santos *et al.*, Phys. Rev. Lett. **98**, 235001 (2007).
[26] L. Lancia *et al.*, Phys. Rev. Lett. **104**, 025001 (2010).
[27] L. Lancia *et al.*, Phys. Rev. Lett. **116**, 075001 (2016).
[28] E. Guillaume *et al.*, High Power Las. Sci. Eng. **2**, e33 (2014).
[29] J.-R. Marquès *et al.*, Phys. Rev. X **9**, 021008 (2019).
[30] R. Kirkwood *et al.*, Nat. Phys. **14**, 80 (2018).
[31] D. W. Forslund, J. M. Kindel and E. L. Lindman, Phys. Fluids **18**, 1002 (1975).
[32] J. F. Drake *et al.*, Phys. Fluids **17**, 778 (1974).
[33] V.M. Malkin, G. Shvets and N.J. Fisch, Phys. Rev. Lett. **82**, 4448 (1999).
[34] W. L. Kruer, *The Physics of Laser Plasma Interactions* (Addison-Wesley, 1988).
[35] J. M. Dawson, Phys. Rev. **113**, 383 (1959).
[36] A. A. Balakin, G. M. Fraiman, N. J. Fisch, and V. M. Malkin, Phys. Plasmas **10**, 4856 (2003).
[37] A. A. Balakin, G. M. Fraiman and I. Y. Dodin, Phys. Plasmas **22**, 053112 (2015).
[38] V. M. Malkin, G. Shvets and N. J. Fisch, Phys. Plasmas **7**, 2232 (2000).
[39] A. A. Balakin, G. M. Fraiman, Q. Jia and N. J. Fisch, Phys. Plasmas **25**, 063106 (2018).
[40] V. M. Malkin and N. J. Fisch, Eur. Phys. J. Special Topics **223**, 1157 (2014).

[41] Z. Toroker, V. M. Malkin and N. J. Fisch, Phys. Plasmas **21**, 113110 (2014).
[42] C. R. Menyuk, D. Levi, and P. Winternitz, Phys. Rev. Lett. **69**, 3048 (1992).
[43] I. Y. Dodin, G. M. Fraiman, V. M. Malkin, and N. J. Fisch, Zh. Eksp. Teor. Fiz. **122**, 723 (2002) [JETP **95**, 625 (2002)].
[44] V. M. Malkin and N. J. Fisch, Phys. Rev. E **80**, 046409 (2009).
[45] R. D. Milroy, C. E. Capjack, and C. R. James, Phys. Fluids **22**, 1922 (1979).
[46] A.A. Andreev and A.N. Sutyagin, Kvant. Elektron. **16**, 2457 (1989) [Sov. J. Quantum Electron. **19**, 1579 (1989)].
[47] B.I. Cohen and C.E. Max, Physics of Fluids **22**, 1115 (1979).
[48] B.I. Cohen *et al.*, Phys. Plasmas **8**, 571 (2001).
[49] E.A. Williams *et al.*, Phys. Plasmas **11**, 231 (2004).
[50] S. Hüller, P. Mulser and A. M. Rubenchik, Phys. Fluids B **3**, 3339 (1991).
[51] F. Schluck *et al.*, Phys. Plasmas **23**, 083105 (2016).
[52] A.A. Andreev *et al.*, Phys. Plasmas **13**, 053110 (2006).
[53] G. Lehmann and K. H. Spatschek, Phys. Plasmas **20**, 073112 (2013).
[54] V. M. Malkin, G. Shvets and N. J. Fisch, Phys. Rev. Lett. **84**,1208 (2000).
[55] J.P. Verboncoeur, A.B. Langdon, and N.T. Gladd, Comput. Phys. Commun. **87**, 199 (1995).
[56] T. P. Coffey, Phys. Fluids **14**, 1402 (1971).
[57] E. Ott, W. H. Manheimer, and H. H. Klein, Phys. Fluids **17**, 1757 (1974).
[58] D. Bohm and E.P. Gross, Phys. Rev. **75**, 1851 (1949).
[59] L. D. Landau, J. Phys (USSR) **10**, 25 (1946) [JETP **16**, 574 (1946)].
[60] J. Dawson, Physics of Fluids **4**, 869 (1961).
[61] M. S. Hur *et al.*, Phys. Rev. Lett. **95**, 115003 (2005).
[62] H. X. Vu, D. F. Dubois and B. Bezzerides, Phys. Rev. Lett. **86**, 4306 (2001).
[63] H. X. Vu, D. F. Dubois and B. Bezzerides, Phys. Plasmas **9**, 1745 (2002).
[64] N. A. Yampolsky and N. J. Fisch, Phys. Plasmas **16**, 072105 (2009).
[65] R. Trines *et al.*, Phys. Rev. Lett. **107**, 105002 (2011).
[66] P. Mardahl *et al.*, Phys. Lett. A **296**, 109 (2002).
[67] G. M. Fraiman, N. A. Yampolsky, V. M. Malkin, and N. J. Fisch, Phys. Plasmas **9**, 3617 (2002).
[68] A. A. Balakin *et al.*, IEEE Trans. Plasma Sci. **33**, 488–489 (2005).
[69] M. S. Hur and J. Wurtele, Comp. Phys. Comm. **180**, 651 (2009).
[70] R. M. G. M. Trines *et al.*, Nature Physics **7**, 87 (2011).
[71] G. Lehmann and K. H. Spatschek, Phys. Plasmas **21**, 053101 (2014).
[72] J. Vieira, R.M.G.M. Trines *et al.*, Nature Communications **7**, 10371 (2016).
